# Robust Estimation for Bivariate Poisson INGARCH Models

**DOI:** 10.3390/e23030367

**Published:** 2021-03-19

**Authors:** Byungsoo Kim, Sangyeol Lee, Dongwon Kim

**Affiliations:** 1 Department of Statistics, Yeungnam University, Gyeongsan 38541, Korea; 2 Department of Statistics, Seoul National University, Seoul 08826, Korea; sylee@stats.snu.ac.kr (S.L.); dongwon.k@snu.ac.kr (D.K.)

**Keywords:** integer-valued time series, bivariate Poisson INGARCH model, outliers, robust estimation, minimum density power divergence estimator

## Abstract

In the integer-valued generalized autoregressive conditional heteroscedastic (INGARCH) models, parameter estimation is conventionally based on the conditional maximum likelihood estimator (CMLE). However, because the CMLE is sensitive to outliers, we consider a robust estimation method for bivariate Poisson INGARCH models while using the minimum density power divergence estimator. We demonstrate the proposed estimator is consistent and asymptotically normal under certain regularity conditions. Monte Carlo simulations are conducted to evaluate the performance of the estimator in the presence of outliers. Finally, a real data analysis using monthly count series of crimes in New South Wales and an artificial data example are provided as an illustration.

## 1. Introduction

Integer-valued time series models have received widespread attention from researchers and practitioners, due to their versatile applications in many scientific areas, including finance, insurance, marketing, and quality control. Numerous studies focus on integer-valued autoregressive (INAR) models to analyze the time series of counts, see Weiß [1] and Scotto et al. [2] for general reviews. Taking a different approach, Ferland et al. [3] proposed using Poisson integer-valued generalized autoregressive conditional heteroscedastic (INGARCH) models and Fokianos et al. [4] developed Poisson AR models to generalize the linear assumption on INGARCH models. The Poisson assumption on INGARCH models has been extended to negative binomial INGARCH models (Davis and Wu [5] and Christou and Fokianos [6]), zero-inflated generalized Poisson INGARCH models (Zhu [7,8] and Lee et al. [9]), and one-parameter exponential family AR models (Davis and Liu [10]). We refer to the review papers by Fokianos [11,12] and Tjøstheim [13,14] for more details.

Researchers invested considerable efforts to extend the univariate integer-valued time series models to bivariate (multivariate) models. For INAR type models, Quoreshi [15] proposed bivariate integer-valued moving average models and Pedeli and Karlis [16] introduced bivariate INAR models with Poisson and negative binomial innovations. Liu [17] proposed bivariate Poisson INGARCH models with a bivariate Poisson distribution that was constructed via the trivariate reduction method and established the stationarity and ergodicity of the model. Andreassen [18] later verified the consistency of the conditional maximum likelihood estimator (CMLE) and Lee et al. [19] studied the asymptotic normality of the CMLE and developed the CMLE- and residual-based change point tests. However, this model has the drawback that it can only accommodate positive correlation between two time series of counts. To cope with this issue, Cui and Zhu [20] recently introduced a new bivariate Poisson INGARCH model based on Lakshminarayana et al.’s [21] bivariate Poisson distribution. Their model can deal with positive or negative correlation, depending on the multiplicative factor parameter. They employed the CMLE for parameter estimation. However, because the CMLE is unduly influenced by outliers, the robust estimation in bivariate Poisson INGARCH models is crucial and deserves thorough investigation.

As such, here we develop a robust estimator for Cui and Zhu’s [20] bivariate Poisson INGARCH models. Among the robust estimation methods, we employ the minimum density power divergence estimator (MDPDE) approach that was originally proposed by Basu et al. [22], because it is well known to consistently provide robust estimators in various situations. For previous works in the context of time series of counts, see Kang and Lee [23], Kim and Lee [24,25], Diop and Kengne [26], Kim and Lee [27], and Lee and Kim [28], who studied the MDPDE for Poisson AR models, zero-inflated Poisson AR models, one-parameter exponential family AR models, and change point tests. For another robust estimation approach in INGARCH models, see Xiong and Zhu [29] and Li et al. [30], who studied Mallows’ quasi-likelihood method. To the best of our knowledge, the robust estimation method for bivariate Poisson INGARCH models has not been previously studied. In earlier studies, the MDPDE was proven to possess strong robust properties against outliers with little loss in asymptotic efficiency relative to the CMLE. This study confirms the same conclusion for bivariate Poisson INGARCH models.

The rest of this paper is organized, as follows. Section 2 constructs the MDPDE for bivariate Poisson INGARCH models. Section 3 shows the asymptotic properties of the MDPDE. Section 4 conducts empirical studies to evaluate the performance of the MDPDE. Section 5 provides concluding remarks. Appendix A provides the proof.

## 2. MDPDE for Bivariate Poisson Ingarch Models

Basu et al. [22] defined the density power divergence $d_{\alpha }$ between two densities *f* and *g*, with a tuning parameter α, as

$$\begin{array}{c} d_{\alpha }\left(g,f\right)=\left\{\begin{array}{cc} \int \{f^{1+\alpha }\left(y\right)-\left(1+\frac{1}{\alpha }\right)g\left(y\right)f^{\alpha }\left(y\right)+\frac{1}{\alpha }g^{1+\alpha }\left(y\right)\}dy, & \alpha >0,\\ \int g(y)(\log g(y)-\log f(y))dy, & \alpha =0. \end{array}\right. \end{array}$$

For a parametric family $\left\{F_{\theta };\;\theta \in \Theta \right\}$ having densities $\left\{f_{\theta }\right\}$ and a distribution *G* with density *g*, they defined the minimum density power divergence functional $T_{\alpha }\left(G\right)$ by $d_{\alpha }\left(g,f_{T_{\alpha }\left(G\right)}\right)=\min_{\theta \in \Theta }d_{\alpha }\left(g,f_{\theta }\right)$. If *G* belongs to $\left\{F_{\theta }\right\}$, which is, $G=F_{\theta^{0}}$ for some $\theta^{0}\in \Theta$, then $T_{\alpha }\left(F_{\theta^{0}}\right)=\theta^{0}$. Let *g* be the density function of a random sample $Y_{1},\ldots ,Y_{n}$. Using the empirical distribution $G_{n}$ to approximate *G*, Basu et al. [22] defined the MDPDE by

$$\begin{array}{c} {\hat{\theta }}_{\alpha ,n}=\underset{\theta \in \Theta }{\mathrm{argmin}}H_{\alpha ,n}\left(\theta \right), \end{array}$$

where $H_{\alpha ,n}\left(\theta \right)=\frac{1}{n}\sum_{t=1}^{n}h_{\alpha ,t}\left(\theta \right)$ and

$$\begin{array}{c} h_{\alpha ,t}\left(\theta \right)=\left\{\begin{array}{cc} \int f_{\theta }^{1+\alpha }\left(y\right)dy-\left(1+\frac{1}{\alpha }\right)f_{\theta }^{\alpha }\left(Y_{t}\right), & \alpha >0,\\ -\log f_{\theta }\left(Y_{t}\right), & \alpha =0. \end{array}\right. \end{array}$$

The tuning parameter α controls the trade-off between the robustness and asymptotic efficiency of the MDPDE. Namely, relatively large α values improve the robustness but the estimator’s efficiency decreases. The MDPDE with α=0 and 1 leads to the MLE and $L_{2}$-distance estimator, respectively. Basu et al. [22] showed the consistency and asymptotic normality of the MDPDE and demonstrated that the estimator is robust against outliers, but it still retains high efficiency when the true distribution belongs to a parametric family $\left\{F_{\theta }\right\}$ and α is close to zero.

We need to define the conditional version of the MDPDE in order to apply the above procedure to bivariate Poisson INGARCH models. Let $\left\{f_{\theta }\left(\cdot |F_{t-1}\right)\right\}$ denote the parametric family of autoregressive models, being indexed by the parameter θ, and let $f_{\theta^{0}}\left(\cdot |F_{t-1}\right)$ be the true conditional density of the time series $Y_{t}$ given $F_{t-1}$, where $F_{t-1}$ is a σ-field generated by $Y_{t-1},Y_{t-2},\ldots$. Subsequently, the MDPDE of $\theta^{0}$ is given by

$$\begin{array}{c} {\hat{\theta }}_{\alpha ,n}=\underset{\theta \in \Theta }{\mathrm{argmin}}H_{\alpha ,n}\left(\theta \right), \end{array}$$

where $H_{\alpha ,n}\left(\theta \right)=\frac{1}{n}\sum_{t=1}^{n}h_{\alpha ,t}\left(\theta \right)$ and

(1) $$\begin{array}{c} h_{\alpha ,t}\left(\theta \right)=\left\{\begin{array}{cc} \int f_{\theta }^{1+\alpha }\left(y|F_{t-1}\right)dy-\left(1+\frac{1}{\alpha }\right)f_{\theta }^{\alpha }\left(Y_{t}|F_{t-1}\right), & \alpha >0,\\ -\log f_{\theta }\left(Y_{t}|F_{t-1}\right), & \alpha =0 \end{array}\right. \end{array}$$

(cf. Section 2 of Kang and Lee [23]).

Let $Y_{t}={\left(Y_{t,1},Y_{t,2}\right)}^{T}$ be a two-dimensional vector of counts at time *t*, namely, $\left\{Y_{t,1},t\geq 1\right\}$ and $\left\{Y_{t,2},t\geq 1\right\}$ are the two time series of counts under consideration. Liu [17] proposed the bivariate Poisson INGARCH model, as follows

$$\begin{array}{c} Y_{t}|F_{t-1}∼BP^{*}\left(\lambda_{t,1},\lambda_{t,2},\phi \right),\;\;\;\lambda_{t}={\left(\lambda_{t,1},\lambda_{t,2}\right)}^{T}=\omega +A\lambda_{t-1}+BY_{t-1}, \end{array}$$

where $F_{t}$ is the σ-field generated by $Y_{t},Y_{t-1},\ldots$, ϕ≥0, $\omega ={\left(\omega_{1},\omega_{2}\right)}^{T}\in R_{+}^{2}$, $A={\left\{a_{ij}\right\}}_{i,j=1,2}$ and $B={\left\{b_{ij}\right\}}_{i,j=1,2}$ are 2×2 matrices with non-negative entries. $BP^{*}\left(\lambda_{t,1},\lambda_{t,2},\phi \right)$ denotes the bivariate Poisson distribution constructed via the trivariate reduction method, whose probability mass function (PMF) is

P(Yt,1=y1,Yt,2=y2|Ft−1)=e−(λt,1+λt,2−ϕ)(λt,1−ϕ)y1y1!(λt,2−ϕ)y2y2!∑s=0min(y1,y2)y1sy2ss!ϕ(λt,1−ϕ)(λt,2−ϕ)s.

In this model, $Cov\left(Y_{t,1},Y_{t,2}|F_{t-1}\right)=\phi \in \left[0,\min\left(\lambda_{t,1},\lambda_{t,2}\right)\right)$, so that the model has a drawback that it can only deal with positive correlation between two components.

To overcome this defect, Cui and Zhu [20] proposed a new bivariate Poisson INGARCH model using the distribution that was proposed by Lakshminarayana et al. [21]. They considered the model:

(2) $$\begin{array}{c} Y_{t}|F_{t-1}∼BP\left(\lambda_{t,1},\lambda_{t,2},\delta \right),\;\;\;\lambda_{t}={\left(\lambda_{t,1},\lambda_{t,2}\right)}^{T}=\omega +A\lambda_{t-1}+BY_{t-1} \end{array}$$

and $BP(\lambda_{t,1},\lambda_{t,2},\delta )$ is the bivariate Poisson distribution constructed as a product of Poisson marginals with a multiplicative factor, whose PMF is given by

(3) $$\begin{array}{ccc} & & P(Y_{t,1}=y_{1},Y_{t,2}=y_{2}|F_{t-1})\\ & = & \frac{\lambda_{t,1}^{y_{1}}\lambda_{t,2}^{y_{2}}}{y_{1}!y_{2}!}e^{-(\lambda_{t,1}+\lambda_{t,2})}\left\{1+\delta \left(e^{-y_{1}}-e^{-c\lambda_{t,1}}\right)\left(e^{-y_{2}}-e^{-c\lambda_{t,2}}\right)\right\}, \end{array}$$

where $c=1-e^{-1}$. The marginal conditional distribution of $Y_{t,1}$ and $Y_{t,2}$ are Poisson with parameters $\lambda_{t,1}$ and $\lambda_{t,2}$, respectively, and $Cov\left(Y_{t,1},Y_{t,2}|F_{t-1}\right)=\delta c^{2}\lambda_{t,1}\lambda_{t,2}e^{-c(\lambda_{t,1}+\lambda_{t,2})}$. Hence, this model supports positive or negative correlation, depending on the multiplicative factor parameter δ. Cui and Zhu [20] established the stationarity and ergodicity of the model under certain conditions and showed the consistency and asymptotic normality of the CMLE.

In this study, we apply the MDPDE to the model (2). We focus on the case that A is a diagonal matrix, because this simplification can reduce the number of model parameters and makes it easy to use in practice, as Heinen and Rengifo [31] suggested. Further, the diagonal setup of A eases the verification of the asymptotic properties of the MDPDE. Similar approaches can be found in Liu [17], Lee et al. [19], and Cui et al. [32]. Let $A=diag(a_{1},a_{2})$. Subsequently, we set $\theta ={\left(\theta_{1}^{T},\theta_{2}^{T},\delta \right)}^{T}$, where $\theta_{1}={\left(\omega_{1},a_{1},b_{11},b_{12}\right)}^{T}$ and $\theta_{2}={\left(\omega_{2},a_{2},b_{21},b_{22}\right)}^{T}$, and write the true parameter as $\theta^{0}={\left({\theta_{1}^{0}}^{T},{\theta_{2}^{0}}^{T},\delta^{0}\right)}^{T}$, where $\theta_{1}^{0}={\left(\omega_{1}^{0},a_{1}^{0},b_{11}^{0},b_{12}^{0}\right)}^{T}$ and $\theta_{2}^{0}={\left(\omega_{2}^{0},a_{2}^{0},b_{21}^{0},b_{22}^{0}\right)}^{T}$.

Given $Y_{1},\ldots ,Y_{n}$ that is generated from (2), from (1), we obtain the MDPDE of $\theta^{0}$ by

$$\begin{array}{c} {\hat{\theta }}_{\alpha ,n}=\underset{\theta \in \Theta }{\mathrm{argmin}}{\tilde{H}}_{\alpha ,n}\left(\theta \right)=\underset{\theta \in \Theta }{\mathrm{argmin}}\frac{1}{n}\sum_{t=1}^{n}{\tilde{h}}_{\alpha ,t}\left(\theta \right), \end{array}$$

where

(4) $$\begin{array}{cc} {\tilde{h}}_{\alpha ,t}\left(\theta \right) & =\left\{\begin{array}{cc} \sum_{y_{1}=0}^{\infty }\sum_{y_{2}=0}^{\infty }f_{\theta }^{1+\alpha }\left(y|{\tilde{\lambda }}_{t}\right)-\left(1+\frac{1}{\alpha }\right)f_{\theta }^{\alpha }\left(Y_{t}|{\tilde{\lambda }}_{t}\right), & \alpha >0,\\ -\log f_{\theta }\left(Y_{t}|{\tilde{\lambda }}_{t}\right), & \alpha =0, \end{array}\right. \end{array}$$

$f_{\theta }\left(y|\lambda_{t}\right)$ for $y={\left(y_{1},y_{2}\right)}^{T}$ is the conditional PMF in (3), and ${\tilde{\lambda }}_{t}$ is recursively defined by

$$\begin{array}{c} {\tilde{\lambda }}_{t}={\left({\tilde{\lambda }}_{t,1},{\tilde{\lambda }}_{t,2}\right)}^{T}=\omega +A{\tilde{\lambda }}_{t-1}+BY_{t-1},\;\;t\geq 2 \end{array}$$

with an arbitrarily chosen initial value ${\tilde{\lambda }}_{1}$. We also use notations $\lambda_{t}\left(\theta \right)$ and ${\tilde{\lambda }}_{t}\left(\theta \right)$ to denote $\lambda_{t}$ and ${\tilde{\lambda }}_{t}$, respectively, in order to emphasize the role of θ.

## 3. Asymptotic Properties of the MDPDE

In this section, we establish the consistency and asymptotic normality of the MDPDE. Throughout this study, ${∥A∥}_{p}$ denotes the *p*-induced norm of matrix A for 1≤p≤∞ and ${∥x∥}_{p}$ is the *p*-norm of vector x. When p=1 and ∞, ${∥A∥}_{1}=\max_{1\leq j\leq n}\sum_{i=1}^{m}\left|a_{ij}\right|$ and ${∥A∥}_{\infty }=\max_{1\leq i\leq m}\sum_{j=1}^{n}\left|a_{ij}\right|$ for $A={\left\{a_{ij}\right\}}_{1\leq i\leq m,1\leq j\leq n}$, respectively. E(·) is taken under $\theta^{0}$. We assume that the following conditions hold in order to verify the asymptotic properties of the MDPDE.

**(A1)**  $\theta_{1}^{0},\;\theta_{2}^{0}$, and $\delta^{0}$ are interior points in the compact parameter spaces $\Theta_{1}$, $\Theta_{2}$, and $\Theta_{3}$, respectively, and $\Theta =\Theta_{1}\times \Theta_{2}\times \Theta_{3}$. In addition, there exist positive constants $\omega_{L}$, $\omega_{U}$, $a_{L}$, $a_{U}$, $b_{L}$, $b_{U}$, and $\delta_{U}$, such that for i,j=1,2,
  $$\begin{array}{c} 0<\omega_{L}\leq \omega_{i}\leq \omega_{U},\;0<a_{L}\leq a_{i}\leq a_{U},\;0<b_{L}\leq b_{ij}\leq b_{U},\;\mathrm{and}\;\left|\delta \right|\leq \delta_{U}. \end{array}$$
**(A2)**  There exist positive constants $\varphi_{L}$ and $\varphi_{U}$ such that for $y={\left(y_{1},y_{2}\right)}^{T}\in N_{0}^{2},\;\lambda ={\left(\lambda_{1},\lambda_{2}\right)}^{T}\in {\left(0,\infty \right)}^{2}$, and $\delta \in \Theta_{3}$,
  $$\begin{array}{c} 0<\varphi_{L}\leq \varphi \left(y,\lambda ,\delta \right)\leq \varphi_{U},\;\mathrm{where}\;\varphi \left(y,\lambda ,\delta \right)=1+\delta \left(e^{-y_{1}}-e^{-c\lambda_{1}}\right)\left(e^{-y_{2}}-e^{-c\lambda_{2}}\right). \end{array}$$
**(A3)**  There exists a p∈[1,∞] such that ${∥A∥}_{p}+2^{\left(1-1/p\right)}{∥B∥}_{p}<1$.

**Remark** **1.** *These conditions can be found in Cui and Zhu [20]. According to Theorem 1 in their study, $\left\{\left(Y_{t},\lambda_{t}\right)\right\}$ is stationary and ergodic under (A1) and (A3).*

Subsequently, we obtain the following results; the proofs are provided in the Appendix A.

**Theorem** **1.** *Under the conditions* ***(A1)*** *–* ***(A3)*** *,*

$$\begin{array}{c} {\hat{\theta }}_{\alpha ,n}\overset{a.s.}{⟶}\theta^{0}\;\;as\;\;n\to \infty . \end{array}$$

**Theorem** **2.** *Under the conditions* ***(A1)*** *–* ***(A3)*** *,*

$$\begin{array}{c} \sqrt{n}\left({\hat{\theta }}_{\alpha ,n}-\theta^{0}\right)\overset{d}{⟶}N\left(0,J_{\alpha }^{-1}K_{\alpha }J_{\alpha }^{-1}\right)\;\;as\;\;n\to \infty , \end{array}$$

*where*

$$\begin{array}{c} J_{\alpha }=-E\left(\frac{\partial^{2}h_{\alpha ,t}\left(\theta^{0}\right)}{\partial \theta \partial \theta^{T}}\right),\;K_{\alpha }=E\left(\frac{\partial h_{\alpha ,t}\left(\theta^{0}\right)}{\partial \theta }\frac{\partial h_{\alpha ,t}\left(\theta^{0}\right)}{\partial \theta^{T}}\right), \end{array}$$

*and $h_{\alpha ,t}\left(\theta \right)$ is defined by replacing ${\tilde{\lambda }}_{t}\left(\theta \right)$ with $\lambda_{t}\left(\theta \right)$ in (4).*

**Remark** **2.** *Because the tuning parameter α controls the trade-off between the robustness and asymptotic efficiency, choosing the optimal α is an important issue in practice. Several researchers investigated the selection criterion of optimal α; see Fujisawa and Eguchi [33], Durio and Isaia [34], and Toma and Broniatowski [35]. Among them, we adopt the method of Warwick [36] to choose α that minimizes the trace of the estimated asymptotic mean squared error ($\hat{AMSE}$) defined by*

$$\hat{AMSE}=\left({\hat{\theta }}_{\alpha ,n}-{\hat{\theta }}_{1,n}\right){\left({\hat{\theta }}_{\alpha ,n}-{\hat{\theta }}_{1,n}\right)}^{T}+\hat{As.var}\left({\hat{\theta }}_{\alpha ,n}\right),$$

*where ${\hat{\theta }}_{1,n}$ is the MDPDE with α=1 and $\hat{As.var}\left({\hat{\theta }}_{\alpha ,n}\right)$ is an estimate of the asymptotic variance of ${\hat{\theta }}_{\alpha ,n}$, which is computed as*

$$\hat{As.var}\left({\hat{\theta }}_{\alpha ,n}\right)={\left(\sum_{t=1}^{n}\frac{\partial^{2}{\tilde{h}}_{\alpha ,t}\left({\hat{\theta }}_{\alpha ,n}\right)}{\partial \theta \partial \theta^{T}}\right)}^{-1}\left(\sum_{t=1}^{n}\frac{\partial {\tilde{h}}_{\alpha ,t}\left({\hat{\theta }}_{\alpha ,n}\right)}{\partial \theta }\frac{\partial {\tilde{h}}_{\alpha ,t}\left({\hat{\theta }}_{\alpha ,n}\right)}{\partial \theta^{T}}\right){\left(\sum_{t=1}^{n}\frac{\partial^{2}{\tilde{h}}_{\alpha ,t}\left({\hat{\theta }}_{\alpha ,n}\right)}{\partial \theta \partial \theta^{T}}\right)}^{-1}.$$

*This criterion is applied to our empirical study in Section 4.2.*

## 4. Empirical Studies

### 4.1. Simulation

In this section, we report the simulation results to evaluate the performance of the MDPDE. The simulation settings are described, as follows. Using the inverse transformation sampling method (cf. Section 2.3 of Verges [37]), we generate $Y_{1},\ldots ,Y_{n}$ from (2) with the initial value $\lambda_{1}={\left(0,0\right)}^{T}$. For the estimation, ${\tilde{\lambda }}_{1}$ is set to be the sample mean of the data. We first consider $\theta ={\left(\omega_{1},a_{1},b_{11},b_{12},\omega_{2},a_{2},b_{21},b_{22},\delta \right)}^{T}={\left(1,0.2,0.1,0.2,0.5,0.3,0.4,0.2,0.5\right)}^{T}$, which satisfies **(A3)** with p=1. In this simulation, we compare the performance of the MDPDE with α>0 with that of the CMLE (α=0). We examine the sample mean, variance, and mean squared error (MSE) of the estimators. The sample size under consideration is n=1000 and the number of repetitions for each simulation is 1000. In Table 1, Table 2, Table 3, Table 4, Table 5, Table 6, Table 7, Table 8, Table 9, Table 10, Table 11, Table 12, Table 13, Table 14, Table 15 and Table 16, the symbol * represents the minimal MSEs for each parameter.

Table 1 indicates that, when the data are not contaminated by outliers, the CMLE exhibits minimal MSEs for all parameters, and the MSEs of the MDPDE with small α are close to those of the CMLE. The MSE of the MDPDE shows an increasing tendency as α increases. Hence, we can conclude that the CMLE outperforms the MDPDE in the absence of outliers.

Now, we consider the situation that the data are contaminated by outliers. To this end, we generate contaminated data $Y_{c,t}={\left(Y_{c,t,1},Y_{c,t,2}\right)}^{T}$ when considering

$$\begin{array}{c} Y_{c,t,i}=Y_{t,i}+P_{t,i}Y_{o,t,i},\;\;i=1,2, \end{array}$$

where $Y_{t,i}$ are generated from (2), $P_{t,i}$ are i.i.d. Bernoulli random variables with success probability *p*, and $Y_{o,t,i}$ are i.i.d. Poisson random variables with mean γ. We consider three cases: (p,γ)=(0.03,5),(0.03,10), and (0.05,10). Table 2, Table 3 and Table 4 report the results. In the tables, the MDPDE appears to have smaller MSEs than the CMLE for all cases, except for the case of α=1 when (p,γ)=(0.03,5). As *p* or γ increases, the MSEs of the CMLE increase faster than those of the MDPDE, which indicates that the MDPDE outperforms the CMLE, as the data are more contaminated by outliers. Moreover, as *p* or γ increases, the symbol ${}^{*}$ tends to move downward. This indicates that, when the data are severely contaminated by outliers, the MDPDE with large α performs better.

We also consider smaller sample size n=200. The results are presented in Table 5, Table 6, Table 7 and Table 8 and they show results similar to those in Table 1, Table 2, Table 3 and Table 4. The variances and MSEs of both the CMLE and MDPDE are larger than those in Table 1, Table 2, Table 3 and Table 4.

In order to evaluate the performance of the MDPDE for negatively cross-correlated data, we consider $\theta ={\left(\omega_{1},a_{1},b_{11},b_{12},\omega_{2},a_{2},b_{21},b_{22},\delta \right)}^{T}={\left(0.5,0.1,0.2,0.4,0.3,0.3,0.2,0.1,-0.4\right)}^{T}$ with the same *p* and γ, as above. The results are reported in Table 9, Table 10, Table 11, Table 12, Table 13, Table 14, Table 15 and Table 16 for n=1000 and 200, respectively. These tables exhibit results that are similar to those in Table 1, Table 2, Table 3, Table 4, Table 5, Table 6, Table 7 and Table 8. Overall, our findings strongly support the assertion that the MDPDE is a functional tool for yielding a robust estimator for bivariate Poisson INGARCH models in the presence of outliers.

### 4.2. Illustrative Examples

**Figure 1:**
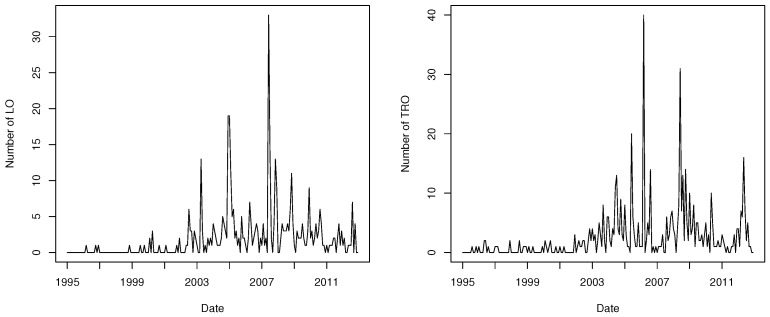
Monthly count series of liquor offences (LO) (**left**) and transport regulatory offences (TRO) (**right**) in Botany Bay.

**Figure 2:**
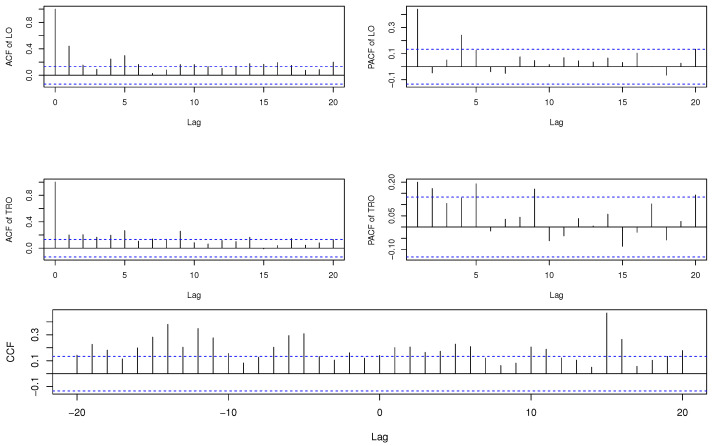
Autocorrelation function (ACF) and partial autocorrelation function (PACF) of LO (**top**) and TRO (**middle**), and cross-correlation function (CCF) (**bottom**) between two series.

First, we illustrate the proposed method by examining the monthly count series of crimes provided by the New South Wales Police Force in Australia. The data set is classified by local government area and offence type. This data set has been studied in many literatures, including Lee et al. [9], Chen and Lee [38,39], Kim and Lee [24], and Lee et al. [40]. To investigate the behavior of the MDPDE in the presence of outliers, we consider the data series of liquor offences (LO) and transport regulatory offences (TRO) in Botany Bay from January 1995 to December 2012, which has 216 observations in each series. Figure 1 plots the monthly count series of LO and TRO and it shows the presence of some deviant observations in each series. The sample mean and variance are 1.912 and 13.14 for LO, and 2.463 and 20.41 for TRO. A large value of the variance of each series is expected to be influenced by outliers. The autocorrelation function (ACF) and partial autocorrelation function (PACF) of LO and TRO, as well as cross-correlation function (CCF) between two series, are given in Figure 2, indicating that the data are both serially and crossly correlated. The cross-correlation coefficient between two series is 0.141.

We fit the model (2) to the data using both the CMLE and the MDPDE. ${\tilde{\lambda }}_{1}$ is set to be the sample mean of the data. Table 17 reports the estimated parameters with various α. The standard errors are given in parentheses and the symbol ${}^{•}$ represents the minimal $\hat{\mathrm{AMSE}}$ provided in Remark 2. In the table, we can observe that the MDPDE has smaller $\hat{\mathrm{AMSE}}$ than the CMLE and the optimal α is chosen to be 0.1. The MDPDE with optimal α is quite different from the CMLE, in particular, $\hat{\delta }$ is about half of the CMLE. This result indicates that outliers can seriously affect the parameter estimation and, thus, the robust estimation method is required when the data are contaminated by outliers.

We clean the data by using the approach that was introduced by Fokianos and Fried [41] and apply the CMLE and the MDPDE to this data in order to illustrate the behavior of the estimators in the absence of outliers. Table 18 reports the results. The standard errors and $\hat{\mathrm{AMSE}}$ tend to decrease compared to Table 17. The CMLE has minimal $\hat{\mathrm{AMSE}}$ and the MDPDE with small α appears to be similar to the CMLE.

Now, we consider an artificial example that has negative cross-correlation coefficient. Following Cui and Zhu [20], we generate 1000 samples from univariate Poisson INGARCH model, i.e.,

$$\begin{array}{c} X_{t}|F_{t-1}∼P\left(\lambda_{t}\right),\;\;\lambda_{t}=1+0.35\lambda_{t-1}+0.45X_{t-1}, \end{array}$$

where $P(\lambda_{t})$ denotes the Poisson distribution with mean $\lambda_{t}$. Further, we observe the contaminated data $X_{c,t}$ as follows

$$\begin{array}{c} X_{c,t}=X_{t}+P_{t}X_{o,t}, \end{array}$$

where $P_{t}$ are i.i.d. Bernoulli random variables with a success probability of 0.03 and $X_{o,t}$ are i.i.d. Poisson random variables with mean 5. Let $Y_{t}={\left(Y_{t,1},Y_{t,2}\right)}^{T}$, where $Y_{t,1}=X_{c,t}$ and $Y_{t,2}=X_{c,t+500}$ for t=1,…,500. The sample mean and variance are 5.196 and 7.380 for $Y_{t,1}$, and 4.538 and 8.129 for $Y_{t,2}$. The cross-correlation coefficient between $Y_{t,1}$ and $Y_{t,2}$ is −0.161. We fit the model (2) to $Y_{t}$ and the results are presented in Table 19. Similar to Table 17, the MDPDE has smaller $\hat{\mathrm{AMSE}}$ than the CMLE. The optimal α is chosen to be 0.3 and the corresponding $\hat{\delta }$ is −0.329, whereas the CMLE is 0.772.

## 5. Concluding Remarks

In this study, we developed the robust estimator for bivariate Poisson INGARCH models based on the MDPDE. In practice, this is important, because outliers can strongly affect the CMLE, which is commonly employed for parameter estimation in INGARCH models. We proved that the MDPDE is consistent and asymptotically normal under regularity conditions. Our simulation study compared the performances of the MDPDE and the CMLE, and confirmed the superiority of the proposed estimator in the presence of outliers. The real data analysis also confirmed the validity of our method as a robust estimator in practice. Although we focused on Cui and Zhu’s [20] bivariate Poisson INGARCH models here, the MDPDE method can be extended to other bivariate INGARCH models. For example, one can consider the copula-based bivariate INGARCH models that were studied by Heinen and Rengifo [42], Andreassen [18], and Fokianos et al. [43]. We leave this issue of extension as our future research.

## Figures and Tables

**Table 1:** Sample mean, variance, and mean squared error (MSE) of estimators when $\theta ={\left(1,0.2,0.1,0.2,0.5,0.3,0.4,0.2,0.5\right)}^{T}$, n=1000, and no outliers exist.

α		${\hat{\omega }}_{1}$	${\hat{a}}_{1}$	${\hat{b}}_{11}$	${\hat{b}}_{12}$	${\hat{\omega }}_{2}$	${\hat{a}}_{2}$	${\hat{b}}_{21}$	${\hat{b}}_{22}$	$\hat{\delta }$
0(CMLE)	Mean	1.010	0.198	0.099	0.199	0.510	0.298	0.401	0.198	0.583
	Var $\times 10^{2}$	3.421	1.062	0.105	0.083	1.344	0.366	0.119	0.100	15.54
	MSE $\times 10^{2}$	3.429 *	1.061 *	0.105 *	0.083 *	1.352 *	0.366 *	0.119 *	0.100 *	16.22 *
0.1	Mean	1.012	0.198	0.099	0.199	0.510	0.297	0.401	0.199	0.577
	Var $\times 10^{2}$	3.527	1.091	0.108	0.083	1.379	0.372	0.121	0.103	15.83
	MSE $\times 10^{2}$	3.537	1.091	0.108	0.084	1.387	0.372	0.121	0.103	16.41
0.2	Mean	1.013	0.197	0.099	0.199	0.510	0.297	0.401	0.199	0.572
	Var $\times 10^{2}$	3.671	1.134	0.113	0.086	1.453	0.387	0.126	0.108	16.42
	MSE $\times 10^{2}$	3.684	1.134	0.113	0.086	1.463	0.388	0.126	0.108	16.92
0.3	Mean	1.013	0.197	0.099	0.199	0.511	0.296	0.401	0.199	0.568
	Var $\times 10^{2}$	3.870	1.195	0.120	0.090	1.555	0.410	0.133	0.114	17.22
	MSE $\times 10^{2}$	3.883	1.195	0.120	0.090	1.565	0.411	0.133	0.114	17.66
0.5	Mean	1.012	0.197	0.100	0.199	0.511	0.294	0.402	0.200	0.559
	Var $\times 10^{2}$	4.336	1.340	0.137	0.101	1.817	0.469	0.151	0.130	19.51
	MSE $\times 10^{2}$	4.347	1.340	0.137	0.101	1.828	0.472	0.152	0.130	19.84
1	Mean	1.007	0.198	0.101	0.200	0.513	0.289	0.405	0.203	0.544
	Var $\times 10^{2}$	6.094	1.864	0.198	0.148	2.805	0.690	0.222	0.189	29.18
	MSE $\times 10^{2}$	6.094	1.863	0.198	0.148	2.818	0.701	0.224	0.190	29.35

**Table 2:** Sample mean, variance, and MSE of estimators when $\theta ={\left(1,0.2,0.1,0.2,0.5,0.3,0.4,0.2,0.5\right)}^{T}$, n=1000, and (p,γ)=(0.03,5).

α		${\hat{\omega }}_{1}$	${\hat{a}}_{1}$	${\hat{b}}_{11}$	${\hat{b}}_{12}$	${\hat{\omega }}_{2}$	${\hat{a}}_{2}$	${\hat{b}}_{21}$	${\hat{b}}_{22}$	$\hat{\delta }$
0(CMLE)	Mean	1.073	0.266	0.077	0.167	0.650	0.339	0.325	0.176	0.728
	Var $\times 10^{2}$	6.363	1.707	0.109	0.105	2.333	0.553	0.168	0.115	17.16
	MSE $\times 10^{2}$	6.897	2.140	0.160	0.213	4.577	0.704	0.736	0.170	22.36
0.1	Mean	1.028	0.264	0.080	0.170	0.607	0.335	0.331	0.179	0.697
	Var $\times 10^{2}$	5.299	1.510	0.098	0.097	2.040	0.512	0.160	0.108	17.23
	MSE $\times 10^{2}$	5.375	1.915	0.139	0.188	3.185	0.635	0.636	0.151	21.09
0.2	Mean	1.008	0.261	0.081	0.171	0.587	0.331	0.335	0.181	0.679
	Var $\times 10^{2}$	5.114	1.491	0.098	0.097	2.031	0.526	0.165	0.110	17.70
	MSE $\times 10^{2}$	5.116 *	1.855	0.133	0.179	2.789	0.621 *	0.583	0.147 *	20.87 *
0.3	Mean	1.000	0.257	0.083	0.172	0.578	0.327	0.339	0.182	0.662
	Var $\times 10^{2}$	5.182	1.526	0.101	0.100	2.099	0.558	0.177	0.115	18.34
	MSE $\times 10^{2}$	5.177	1.846 *	0.131 *	0.177 *	2.701 *	0.628	0.548	0.148	20.95
0.5	Mean	0.997	0.248	0.086	0.174	0.572	0.317	0.346	0.184	0.633
	Var $\times 10^{2}$	5.729	1.682	0.114	0.116	2.381	0.658	0.220	0.136	20.02
	MSE $\times 10^{2}$	5.724	1.910	0.134	0.183	2.899	0.686	0.516	0.162	21.77
1	Mean	1.007	0.230	0.094	0.179	0.578	0.296	0.363	0.191	0.587
	Var $\times 10^{2}$	7.297	2.213	0.166	0.168	3.435	0.965	0.315	0.205	29.90
	MSE $\times 10^{2}$	7.294	2.301	0.170	0.210	4.039	0.966	0.449 *	0.214	30.62

**Table 3:** Sample mean, variance, and MSE of estimators when $\theta ={\left(1,0.2,0.1,0.2,0.5,0.3,0.4,0.2,0.5\right)}^{T}$, n=1000, and (p,γ)=(0.03,10).

α		${\hat{\omega }}_{1}$	${\hat{a}}_{1}$	${\hat{b}}_{11}$	${\hat{b}}_{12}$	${\hat{\omega }}_{2}$	${\hat{a}}_{2}$	${\hat{b}}_{21}$	${\hat{b}}_{22}$	$\hat{\delta }$
0(CMLE)	Mean	1.141	0.349	0.052	0.123	0.846	0.398	0.230	0.141	1.113
	Var $\times 10^{2}$	16.43	3.478	0.101	0.140	5.886	1.087	0.265	0.138	21.91
	MSE $\times 10^{2}$	18.39	5.702	0.335	0.736	17.88	2.051	3.138	0.487	59.51
0.1	Mean	1.015	0.329	0.057	0.131	0.706	0.382	0.248	0.150	0.865
	Var $\times 10^{2}$	7.844	2.031	0.069	0.095	3.087	0.672	0.224	0.100	19.42
	MSE $\times 10^{2}$	7.860	3.703	0.250	0.566	7.329	1.348	2.523	0.355	32.72
0.2	Mean	0.995	0.314	0.060	0.134	0.680	0.365	0.259	0.153	0.802
	Var $\times 10^{2}$	7.073	1.948	0.068	0.095	2.912	0.677	0.244	0.104	19.42
	MSE $\times 10^{2}$	7.068	3.252	0.225	0.529	6.156 *	1.105	2.245	0.321	28.54
0.3	Mean	1.002	0.298	0.064	0.137	0.681	0.349	0.269	0.157	0.765
	Var $\times 10^{2}$	6.995	1.972	0.075	0.102	3.030	0.742	0.280	0.114	19.94
	MSE $\times 10^{2}$	6.989 *	2.936	0.207	0.499	6.287	0.977	2.005	0.301	26.92
0.5	Mean	1.034	0.264	0.072	0.145	0.695	0.314	0.293	0.165	0.706
	Var $\times 10^{2}$	7.365	2.137	0.097	0.125	3.415	0.913	0.382	0.146	21.81
	MSE $\times 10^{2}$	7.475	2.545	0.176	0.430	7.223	0.932 *	1.536	0.266 *	26.01 *
1	Mean	1.088	0.198	0.095	0.167	0.719	0.242	0.353	0.191	0.604
	Var $\times 10^{2}$	7.825	2.377	0.171	0.203	4.553	1.273	0.601	0.258	30.55
	MSE $\times 10^{2}$	8.592	2.375 *	0.173 *	0.309 *	9.328	1.611	0.818${}^{*}$	0.267	31.61

**Table 4:** Sample mean, variance, and MSE of estimators when $\theta ={\left(1,0.2,0.1,0.2,0.5,0.3,0.4,0.2,0.5\right)}^{T}$, n=1000, and (p,γ)=(0.05,10).

α		${\hat{\omega }}_{1}$	${\hat{a}}_{1}$	${\hat{b}}_{11}$	${\hat{b}}_{12}$	${\hat{\omega }}_{2}$	${\hat{a}}_{2}$	${\hat{b}}_{21}$	${\hat{b}}_{22}$	$\hat{\delta }$
0(CMLE)	Mean	1.223	0.404	0.040	0.093	0.990	0.449	0.167	0.114	1.635
	Var $\times 10^{2}$	28.47	4.763	0.086	0.128	11.74	1.691	0.229	0.131	29.21
	MSE $\times 10^{2}$	33.40	8.909	0.442	1.281	35.70	3.897	5.645	0.867	158.1
0.1	Mean	1.012	0.390	0.046	0.103	0.772	0.437	0.185	0.125	1.057
	Var $\times 10^{2}$	11.78	2.695	0.056	0.083	4.883	0.952	0.188	0.095	21.44
	MSE $\times 10^{2}$	11.78	6.291	0.349	1.031	12.27	2.820	4.823	0.661	52.48
0.2	Mean	0.967	0.377	0.048	0.105	0.724	0.421	0.192	0.128	0.935
	Var $\times 10^{2}$	9.531	2.414	0.052	0.080	4.163	0.896	0.203	0.093	20.74
	MSE $\times 10^{2}$	9.633	5.529	0.324	0.986	9.168	2.359	4.525	0.608	39.63
0.3	Mean	0.971	0.361	0.050	0.107	0.720	0.405	0.199	0.131	0.879
	Var $\times 10^{2}$	9.450	2.465	0.055	0.086	4.189	0.962	0.236	0.101	20.90
	MSE $\times 10^{2}$	9.526 *	5.040	0.308	0.953	9.029 *	2.068	4.296	0.578	35.21
0.5	Mean	1.004	0.327	0.056	0.113	0.741	0.369	0.217	0.138	0.801
	Var $\times 10^{2}$	9.878	2.724	0.071	0.112	4.689	1.209	0.363	0.132	22.32
	MSE $\times 10^{2}$	9.870	4.336	0.269	0.861	10.51	1.687 *	3.700	0.511	31.33 *
1	Mean	1.102	0.229	0.084	0.142	0.807	0.257	0.300	0.170	0.651
	Var $\times 10^{2}$	10.28	3.134	0.183	0.238	5.959	1.804	0.946	0.304	30.79
	MSE $\times 10^{2}$	11.32	3.214 *	0.208 *	0.574 *	15.35	1.990	1.936 *	0.392 *	33.03

**Table 5:** Sample mean, variance, and MSE of estimators when $\theta ={\left(1,0.2,0.1,0.2,0.5,0.3,0.4,0.2,0.5\right)}^{T}$, n=200, and no outliers exist.

α		${\hat{\omega }}_{1}$	${\hat{a}}_{1}$	${\hat{b}}_{11}$	${\hat{b}}_{12}$	${\hat{\omega }}_{2}$	${\hat{a}}_{2}$	${\hat{b}}_{21}$	${\hat{b}}_{22}$	$\hat{\delta }$
0(CMLE)	Mean	1.005	0.208	0.089	0.199	0.541	0.281	0.411	0.195	0.893
	Var $\times 10^{2}$	12.41	3.866	0.426	0.394	7.816	2.078	0.651	0.553	71.23
	MSE $\times 10^{2}$	12.40 *	3.869 *	0.437 *	0.394 *	7.973 *	2.112 *	0.663 *	0.555 *	86.57
0.1	Mean	0.975	0.203	0.087	0.193	0.529	0.271	0.400	0.191	0.786
	Var $\times 10^{2}$	14.98	3.919	0.439	0.498	8.317	2.212	1.097	0.649	59.99
	MSE $\times 10^{2}$	15.03	3.916	0.455	0.502	8.392	2.296	1.096	0.658	68.12
0.2	Mean	0.970	0.203	0.087	0.192	0.527	0.267	0.400	0.191	0.756
	Var $\times 10^{2}$	15.48	3.965	0.458	0.520	8.672	2.292	1.176	0.687	60.78
	MSE $\times 10^{2}$	15.55	3.962	0.473	0.526	8.734	2.396	1.174	0.695	67.27 *
0.3	Mean	0.962	0.204	0.088	0.191	0.525	0.263	0.400	0.191	0.730
	Var $\times 10^{2}$	16.41	4.166	0.477	0.555	9.040	2.366	1.274	0.734	63.50
	MSE $\times 10^{2}$	16.54	4.163	0.492	0.563	9.096	2.497	1.273	0.741	68.71
0.5	Mean	0.945	0.202	0.088	0.188	0.521	0.254	0.398	0.192	0.685
	Var $\times 10^{2}$	18.64	4.513	0.527	0.653	10.34	2.653	1.561	0.873	70.39
	MSE $\times 10^{2}$	18.93	4.509	0.540	0.666	10.38	2.863	1.560	0.879	73.75
1	Mean	0.968	0.209	0.102	0.204	0.537	0.249	0.433	0.213	0.684
	Var $\times 10^{2}$	18.37	5.307	0.757	0.817	11.99	3.117	1.327	1.159	135.3
	MSE $\times 10^{2}$	18.45	5.310	0.757	0.817	12.12	3.374	1.437	1.175	138.5

**Table 6:** Sample mean, variance and MSE of estimators when $\theta ={\left(1,0.2,0.1,0.2,0.5,0.3,0.4,0.2,0.5\right)}^{T}$, n=200, and (p,γ)=(0.03,5).

α		${\hat{\omega }}_{1}$	${\hat{a}}_{1}$	${\hat{b}}_{11}$	${\hat{b}}_{12}$	${\hat{\omega }}_{2}$	${\hat{a}}_{2}$	${\hat{b}}_{21}$	${\hat{b}}_{22}$	$\hat{\delta }$
0(CMLE)	Mean	1.056	0.276	0.077	0.164	0.662	0.324	0.339	0.173	1.054
	Var $\times 10^{2}$	20.83	5.501	0.419	0.489	12.34	2.785	0.918	0.619	80.49
	MSE $\times 10^{2}$	21.13	6.078	0.471	0.616 *	14.94	2.839	1.292 *	0.690 *	111.2
0.1	Mean	0.992	0.262	0.077	0.163	0.605	0.311	0.334	0.171	0.925
	Var $\times 10^{2}$	20.38	5.118	0.411	0.510	11.65	2.801	1.153	0.641	67.19
	MSE $\times 10^{2}$	20.37	5.496	0.463 *	0.648	12.75	2.810 *	1.581	0.724	85.15
0.2	Mean	0.973	0.253	0.079	0.165	0.585	0.305	0.338	0.172	0.882
	Var $\times 10^{2}$	19.71	4.993	0.422	0.525	11.55	2.817	1.207	0.652	68.88
	MSE $\times 10^{2}$	19.76	5.265	0.465	0.645	12.26 *	2.816	1.594	0.730	83.37
0.3	Mean	0.958	0.247	0.081	0.165	0.577	0.296	0.340	0.172	0.840
	Var $\times 10^{2}$	19.93	5.028	0.445	0.563	12.33	2.962	1.321	0.690	70.67
	MSE $\times 10^{2}$	20.09	5.244	0.483	0.682	12.90	2.961	1.681	0.766	82.17 *
0.5	Mean	0.944	0.234	0.084	0.167	0.572	0.281	0.344	0.174	0.774
	Var $\times 10^{2}$	20.94	5.080	0.503	0.647	13.53	3.241	1.574	0.806	78.15
	MSE $\times 10^{2}$	21.23	5.193 *	0.528	0.756	14.04	3.273	1.885	0.873	85.55
1	Mean	0.960	0.236	0.101	0.187	0.592	0.266	0.388	0.198	0.770
	Var $\times 10^{2}$	19.00	5.571	0.755	0.859	15.57	3.851	1.689	1.119	147.0
	MSE $\times 10^{2}$	19.14 *	5.696	0.754	0.876	16.40	3.962	1.702	1.119	154.2

**Table 7:** Sample mean, variance, and MSE of estimators when $\theta ={\left(1,0.2,0.1,0.2,0.5,0.3,0.4,0.2,0.5\right)}^{T}$, n=200, and (p,γ)=(0.03,10).

α		${\hat{\omega }}_{1}$	${\hat{a}}_{1}$	${\hat{b}}_{11}$	${\hat{b}}_{12}$	${\hat{\omega }}_{2}$	${\hat{a}}_{2}$	${\hat{b}}_{21}$	${\hat{b}}_{22}$	$\hat{\delta }$
0(CMLE)	Mean	1.128	0.349	0.052	0.126	0.860	0.388	0.241	0.135	1.365
	Var $\times 10^{2}$	38.79	8.126	0.345	0.618	26.05	4.690	1.145	0.746	96.45
	MSE $\times 10^{2}$	40.38	10.33	0.574	1.161	38.95	5.467	3.659	1.174	171.2
0.1	Mean	1.003	0.314	0.054	0.128	0.715	0.355	0.250	0.141	1.050
	Var $\times 10^{2}$	28.42	6.643	0.269	0.507	16.99	3.644	1.158	0.616	69.06
	MSE $\times 10^{2}$	28.39	7.925	0.480	1.021	21.59	3.938	3.403	0.961	99.19
0.2	Mean	0.980	0.296	0.057	0.130	0.679	0.337	0.258	0.146	0.953
	Var $\times 10^{2}$	26.04	6.348	0.270	0.505	15.82	3.612	1.262	0.628	67.71
	MSE $\times 10^{2}$	26.05	7.268	0.455 *	0.991	19.02	3.749	3.268	0.914	88.19
0.3	Mean	0.972	0.289	0.060	0.133	0.678	0.320	0.270	0.151	0.893
	Var $\times 10^{2}$	25.20	6.357	0.299	0.535	15.69	3.649	1.407	0.660	69.05
	MSE $\times 10^{2}$	25.26	7.142	0.457	0.987 *	18.84 *	3.683 *	3.096	0.894 *	84.43
0.5	Mean	0.974	0.264	0.070	0.139	0.673	0.287	0.294	0.160	0.783
	Var $\times 10^{2}$	24.72	6.143	0.399	0.643	16.00	3.836	1.847	0.794	75.64
	MSE $\times 10^{2}$	24.76	6.548	0.490	1.019	18.96	3.848	2.963	0.953	83.56 *
1	Mean	1.007	0.232	0.100	0.171	0.677	0.235	0.374	0.200	0.657
	Var $\times 10^{2}$	21.91	6.221	0.778	1.007	16.89	3.717	2.460	1.238	130.0
	MSE $\times 10^{2}$	21.89 *	6.319 *	0.777	1.088	20.01	4.133	2.526 *	1.237	132.3

**Table 8:** Sample mean, variance, and MSE of estimators when $\theta ={\left(1,0.2,0.1,0.2,0.5,0.3,0.4,0.2,0.5\right)}^{T}$, n=200, and (p,γ)=(0.05,10).

α		${\hat{\omega }}_{1}$	${\hat{a}}_{1}$	${\hat{b}}_{11}$	${\hat{b}}_{12}$	${\hat{\omega }}_{2}$	${\hat{a}}_{2}$	${\hat{b}}_{21}$	${\hat{b}}_{22}$	$\hat{\delta }$
0(CMLE)	Mean	1.171	0.406	0.046	0.097	1.041	0.420	0.183	0.108	1.814
	Var $\times 10^{2}$	53.26	9.255	0.326	0.521	45.18	6.131	1.054	0.654	133.0
	MSE $\times 10^{2}$	56.14	13.48	0.619	1.572	74.38	7.569	5.761	1.504	305.4
0.1	Mean	1.037	0.347	0.047	0.102	0.821	0.389	0.192	0.117	1.203
	Var $\times 10^{2}$	36.17	7.578	0.227	0.430	26.89	4.810	1.034	0.549	80.49
	MSE $\times 10^{2}$	36.28	9.719	0.509	1.388	37.17	5.600	5.372	1.244	129.8
0.2	Mean	0.989	0.334	0.049	0.104	0.772	0.370	0.199	0.122	1.064
	Var $\times 10^{2}$	31.43	7.373	0.218	0.421	23.29	4.607	1.106	0.554	77.26
	MSE $\times 10^{2}$	31.41	9.171	0.477	1.344	30.69	5.097	5.144	1.156	108.9
0.3	Mean	0.989	0.320	0.051	0.106	0.762	0.355	0.207	0.126	0.984
	Var $\times 10^{2}$	30.35	7.338	0.234	0.443	22.64	4.685	1.247	0.602	76.99
	MSE $\times 10^{2}$	30.33	8.773	0.472 *	1.327	29.47	4.985	4.985	1.149 *	100.4
0.5	Mean	0.984	0.293	0.058	0.112	0.764	0.314	0.229	0.135	0.855
	Var $\times 10^{2}$	30.12	7.263	0.332	0.558	22.81	4.884	1.791	0.781	80.40
	MSE $\times 10^{2}$	30.12	8.122	0.505	1.331	29.73	4.897	4.726	1.206	92.95 *
1	Mean	1.046	0.239	0.097	0.151	0.774	0.243	0.333	0.178	0.696
	Var $\times 10^{2}$	23.99	6.497	0.805	1.059	21.95	4.517	3.261	1.366	136.2
	MSE $\times 10^{2}$	24.17 *	6.645 *	0.805	1.302 *	29.46 *	4.839 *	3.708 *	1.413	139.9

**Table 9:** Sample mean, variance, and MSE of estimators when $\theta ={\left(0.5,0.1,0.2,0.4,0.3,0.3,0.2,0.1,-0.4\right)}^{T}$, n=1000, and no outliers exist.

α		${\hat{\omega }}_{1}$	${\hat{a}}_{1}$	${\hat{b}}_{11}$	${\hat{b}}_{12}$	${\hat{\omega }}_{2}$	${\hat{a}}_{2}$	${\hat{b}}_{21}$	${\hat{b}}_{22}$	$\hat{\delta }$
0(CMLE)	Mean	0.501	0.103	0.199	0.397	0.306	0.296	0.200	0.098	−0.385
	Var $\times 10^{2}$	0.570	0.508	0.105	0.159	0.518	1.029	0.080	0.108	6.231
	MSE $\times 10^{2}$	0.569 *	0.508 *	0.105 *	0.160 *	0.522 *	1.030 *	0.080 *	0.109 *	6.247 *
0.1	Mean	0.501	0.103	0.199	0.397	0.306	0.295	0.200	0.098	−0.384
	Var $\times 10^{2}$	0.578	0.515	0.107	0.160	0.530	1.040	0.082	0.111	6.347
	MSE $\times 10^{2}$	0.578	0.515	0.108	0.161	0.534	1.041	0.082	0.112	6.367
0.2	Mean	0.501	0.103	0.199	0.397	0.307	0.295	0.200	0.098	−0.383
	Var $\times 10^{2}$	0.600	0.532	0.113	0.166	0.556	1.082	0.086	0.117	6.564
	MSE $\times 10^{2}$	0.600	0.533	0.113	0.167	0.560	1.083	0.086	0.117	6.588
0.3	Mean	0.501	0.104	0.199	0.397	0.307	0.294	0.200	0.098	−0.381
	Var $\times 10^{2}$	0.627	0.554	0.119	0.175	0.591	1.145	0.092	0.124	6.848
	MSE $\times 10^{2}$	0.627	0.555	0.119	0.176	0.595	1.147	0.092	0.125	6.876
0.5	Mean	0.500	0.105	0.198	0.398	0.308	0.292	0.201	0.099	−0.380
	Var $\times 10^{2}$	0.702	0.615	0.137	0.199	0.685	1.320	0.106	0.142	7.577
	MSE $\times 10^{2}$	0.701	0.617	0.137	0.200	0.690	1.325	0.106	0.142	7.610
1	Mean	0.495	0.110	0.198	0.399	0.310	0.287	0.203	0.100	−0.382
	Var $\times 10^{2}$	0.972	0.839	0.201	0.290	0.942	1.864	0.155	0.195	10.09
	MSE $\times 10^{2}$	0.974	0.848	0.201	0.290	0.951	1.878	0.156	0.194	10.12

**Table 10:** Sample mean, variance, and MSE of estimators when $\theta ={\left(0.5,0.1,0.2,0.4,0.3,0.3,0.2,0.1,-0.4\right)}^{T}$, n=1000, and (p,γ)=(0.03,5).

α		${\hat{\omega }}_{1}$	${\hat{a}}_{1}$	${\hat{b}}_{11}$	${\hat{b}}_{12}$	${\hat{\omega }}_{2}$	${\hat{a}}_{2}$	${\hat{b}}_{21}$	${\hat{b}}_{22}$	$\hat{\delta }$
0(CMLE)	Mean	0.633	0.194	0.143	0.269	0.399	0.368	0.147	0.064	−0.097
	Var $\times 10^{2}$	1.794	1.343	0.152	0.263	1.741	2.315	0.125	0.123	5.603
	MSE $\times 10^{2}$	3.560	2.219	0.474	1.974	2.728	2.769	0.409	0.255	14.79
0.1	Mean	0.572	0.186	0.149	0.280	0.350	0.360	0.153	0.067	−0.143
	Var $\times 10^{2}$	1.191	1.013	0.126	0.235	1.047	1.659	0.100	0.094	5.787
	MSE $\times 10^{2}$	1.711	1.743	0.390	1.676	1.297	2.016	0.325	0.205	12.38
0.2	Mean	0.550	0.177	0.151	0.286	0.335	0.350	0.155	0.068	−0.169
	Var $\times 10^{2}$	1.082	0.958	0.124	0.240	0.950	1.608	0.100	0.090	6.076
	MSE $\times 10^{2}$	1.335	1.543	0.361	1.536	1.074 *	1.861 *	0.305	0.191	11.43
0.3	Mean	0.543	0.167	0.154	0.292	0.333	0.340	0.156	0.070	−0.187
	Var $\times 10^{2}$	1.055	0.950	0.129	0.254	0.976	1.706	0.107	0.095	6.375
	MSE $\times 10^{2}$	1.241	1.401	0.344	1.427	1.083	1.868	0.297	0.184	10.89
0.5	Mean	0.542	0.148	0.159	0.304	0.339	0.318	0.161	0.075	−0.214
	Var $\times 10^{2}$	1.050	0.951	0.147	0.290	1.118	2.017	0.125	0.113	7.038
	MSE $\times 10^{2}$	1.229 *	1.185	0.315	1.203	1.270	2.049	0.279	0.175 *	10.49 *
1	Mean	0.548	0.112	0.176	0.340	0.360	0.268	0.176	0.090	−0.247
	Var $\times 10^{2}$	1.136	0.953	0.214	0.399	1.425	2.636	0.188	0.184	9.324
	MSE $\times 10^{2}$	1.363	0.966 *	0.271 *	0.756 *	1.783	2.733	0.244 *	0.194	11.64

**Table 11:** Sample mean, variance, and MSE of estimators when $\theta ={\left(0.5,0.1,0.2,0.4,0.3,0.3,0.2,0.1,-0.4\right)}^{T}$, n=1000, and (p,γ)=(0.03,10).

α		${\hat{\omega }}_{1}$	${\hat{a}}_{1}$	${\hat{b}}_{11}$	${\hat{b}}_{12}$	${\hat{\omega }}_{2}$	${\hat{a}}_{2}$	${\hat{b}}_{21}$	${\hat{b}}_{22}$	$\hat{\delta }$
0(CMLE)	Mean	0.774	0.295	0.087	0.149	0.525	0.415	0.094	0.037	0.336
	Var $\times 10^{2}$	7.976	4.279	0.176	0.346	7.305	6.070	0.187	0.103	7.338
	MSE $\times 10^{2}$	15.50	8.069	1.442	6.644	12.37	7.392	1.303	0.501	61.46
0.1	Mean	0.612	0.254	0.100	0.173	0.373	0.399	0.106	0.040	0.019
	Var $\times 10^{2}$	2.368	2.008	0.104	0.287	1.694	2.548	0.105	0.049	6.426
	MSE $\times 10^{2}$	3.628	4.369	1.109	5.441	2.231	3.521	0.982	0.406	23.94
0.2	Mean	0.596	0.227	0.103	0.182	0.364	0.378	0.108	0.042	−0.035
	Var $\times 10^{2}$	2.029	1.866	0.106	0.336	1.567	2.548	0.107	0.050	6.677
	MSE $\times 10^{2}$	2.944	3.490	1.048	5.081	1.971 *	3.160	0.949	0.384	20.01
0.3	Mean	0.600	0.203	0.108	0.195	0.372	0.355	0.112	0.046	−0.069
	Var $\times 10^{2}$	1.894	1.823	0.122	0.425	1.601	2.697	0.123	0.060	6.909
	MSE $\times 10^{2}$	2.884	2.873	0.973	4.637	2.111	2.997	0.889	0.353	17.86
0.5	Mean	0.611	0.145	0.125	0.240	0.401	0.287	0.130	0.061	−0.135
	Var $\times 10^{2}$	1.518	1.521	0.177	0.691	1.608	2.883	0.181	0.107	7.489
	MSE $\times 10^{2}$	2.744	1.725	0.742	3.259	2.619	2.898 *	0.674	0.259	14.50
1	Mean	0.594	0.059	0.178	0.360	0.440	0.155	0.185	0.107	−0.249
	Var $\times 10^{2}$	0.941	0.570	0.268	0.634	1.291	2.214	0.278	0.216	9.962
	MSE $\times 10^{2}$	1.828 *	0.737 *	0.316 *	0.794 *	3.262	4.327	0.301 *	0.220 *	12.22 *

**Table 12:** Sample mean, variance, and MSE of estimators when $\theta ={\left(0.5,0.1,0.2,0.4,0.3,0.3,0.2,0.1,-0.4\right)}^{T}$, n=1000, and (p,γ)=(0.05,10).

α		${\hat{\omega }}_{1}$	${\hat{a}}_{1}$	${\hat{b}}_{11}$	${\hat{b}}_{12}$	${\hat{\omega }}_{2}$	${\hat{a}}_{2}$	${\hat{b}}_{21}$	${\hat{b}}_{22}$	$\hat{\delta }$
0(CMLE)	Mean	0.870	0.382	0.059	0.086	0.645	0.451	0.062	0.027	0.829
	Var $\times 10^{2}$	17.32	6.408	0.129	0.206	14.69	8.150	0.135	0.073	8.777
	MSE $\times 10^{2}$	30.96	14.37	2.130	10.09	26.58	10.41	2.026	0.604	159.9
0.1	Mean	0.621	0.346	0.070	0.103	0.396	0.446	0.074	0.029	0.170
	Var $\times 10^{2}$	4.575	3.255	0.073	0.158	2.948	3.708	0.076	0.036	6.635
	MSE $\times 10^{2}$	6.034	9.327	1.762	8.971	3.862	5.837	1.659	0.545	39.12
0.2	Mean	0.585	0.327	0.070	0.104	0.370	0.431	0.073	0.029	0.089
	Var $\times 10^{2}$	3.641	2.988	0.065	0.164	2.294	3.311	0.068	0.031	6.911
	MSE $\times 10^{2}$	4.360	8.156	1.749	8.898	2.788 *	5.022	1.670	0.540	30.79
0.3	Mean	0.586	0.311	0.071	0.107	0.374	0.417	0.074	0.029	0.058
	Var $\times 10^{2}$	3.517	3.054	0.072	0.193	2.353	3.531	0.074	0.033	7.089
	MSE $\times 10^{2}$	4.249	7.500	1.727	8.805	2.893	4.895	1.661	0.532	28.06
0.5	Mean	0.608	0.265	0.080	0.124	0.399	0.371	0.083	0.035	0.016
	Var $\times 10^{2}$	3.465	3.335	0.114	0.398	2.559	4.119	0.120	0.055	7.515
	MSE $\times 10^{2}$	4.628	6.044	1.555	8.030	3.537	4.613 *	1.492	0.482	24.83
1	Mean	0.637	0.087	0.148	0.296	0.481	0.161	0.153	0.096	−0.144
	Var $\times 10^{2}$	1.536	1.591	0.410	1.613	1.732	3.105	0.408	0.306	9.724
	MSE $\times 10^{2}$	3.424 *	1.606 *	0.682 *	2.695 *	4.999	5.042	0.626 *	0.308 *	16.28 *

**Table 13:** Sample mean, variance, and MSE of estimators when $\theta ={\left(0.5,0.1,0.2,0.4,0.3,0.3,0.2,0.1,-0.4\right)}^{T}$, n=200, and no outliers exist.

α		${\hat{\omega }}_{1}$	${\hat{a}}_{1}$	${\hat{b}}_{11}$	${\hat{b}}_{12}$	${\hat{\omega }}_{2}$	${\hat{a}}_{2}$	${\hat{b}}_{21}$	${\hat{b}}_{22}$	$\hat{\delta }$
0(CMLE)	Mean	0.487	0.131	0.182	0.394	0.316	0.287	0.203	0.092	−0.313
	Var $\times 10^{2}$	2.173	2.095	0.526	0.806	2.213	4.245	0.411	0.475	33.35
	MSE $\times 10^{2}$	2.187 *	2.186 *	0.558 *	0.809 *	2.237 *	4.257 *	0.412 *	0.481 *	34.07
0.1	Mean	0.483	0.129	0.181	0.390	0.314	0.284	0.202	0.091	−0.294
	Var $\times 10^{2}$	2.391	2.104	0.571	0.958	2.337	4.358	0.452	0.487	31.43
	MSE $\times 10^{2}$	2.416	2.188	0.609	0.967	2.353	4.379	0.452	0.495	32.53 *
0.2	Mean	0.481	0.131	0.180	0.388	0.312	0.283	0.202	0.090	−0.285
	Var $\times 10^{2}$	2.542	2.158	0.608	1.040	2.427	4.490	0.484	0.512	31.25
	MSE $\times 10^{2}$	2.577	2.250	0.649	1.054	2.439	4.513	0.483	0.520	32.55
0.3	Mean	0.479	0.134	0.180	0.388	0.311	0.284	0.202	0.091	−0.284
	Var $\times 10^{2}$	2.612	2.225	0.636	1.069	2.531	4.657	0.503	0.537	32.24
	MSE $\times 10^{2}$	2.653	2.337	0.677	1.082	2.541	4.679	0.503	0.545	33.55
0.5	Mean	0.477	0.137	0.180	0.389	0.313	0.280	0.204	0.092	−0.285
	Var $\times 10^{2}$	2.860	2.423	0.726	1.183	2.681	4.733	0.554	0.601	35.16
	MSE $\times 10^{2}$	2.909	2.555	0.766	1.194	2.695	4.769	0.555	0.606	36.43
1	Mean	0.473	0.145	0.185	0.399	0.314	0.276	0.212	0.100	−0.324
	Var $\times 10^{2}$	3.364	3.057	1.016	1.549	3.003	5.321	0.746	0.858	48.77
	MSE $\times 10^{2}$	3.434	3.252	1.038	1.548	3.021	5.375	0.760	0.857	49.31

**Table 14:** Sample mean, variance, and MSE of estimators when $\theta ={\left(0.5,0.1,0.2,0.4,0.3,0.3,0.2,0.1,-0.4\right)}^{T}$, n=200, and (p,γ)=(0.03,5).

α		${\hat{\omega }}_{1}$	${\hat{a}}_{1}$	${\hat{b}}_{11}$	${\hat{b}}_{12}$	${\hat{\omega }}_{2}$	${\hat{a}}_{2}$	${\hat{b}}_{21}$	${\hat{b}}_{22}$	$\hat{\delta }$
0(CMLE)	Mean	0.611	0.215	0.138	0.270	0.396	0.349	0.157	0.068	−0.056
	Var $\times 10^{2}$	6.324	4.595	0.661	1.242	5.415	6.896	0.600	0.472	28.97
	MSE $\times 10^{2}$	7.555	5.916	1.050	2.927	6.330	7.124	0.786	0.574	40.76
0.1	Mean	0.561	0.202	0.141	0.278	0.348	0.345	0.160	0.068	−0.086
	Var $\times 10^{2}$	4.635	3.815	0.593	1.110	3.799	5.860	0.497	0.380	28.62
	MSE $\times 10^{2}$	5.004	4.853	0.942	2.597	4.023	6.053	0.653	0.483	38.44
0.2	Mean	0.537	0.192	0.142	0.282	0.329	0.338	0.161	0.068	−0.099
	Var $\times 10^{2}$	4.374	3.640	0.598	1.175	3.512	5.797	0.497	0.367	28.89
	MSE $\times 10^{2}$	4.504	4.487	0.930 *	2.562	3.592	5.933 *	0.649 *	0.468 *	37.90 *
0.3	Mean	0.526	0.187	0.144	0.287	0.325	0.330	0.162	0.070	−0.115
	Var $\times 10^{2}$	4.313	3.636	0.619	1.264	3.494	5.913	0.517	0.383	29.94
	MSE $\times 10^{2}$	4.377	4.383	0.932	2.529 *	3.553 *	5.998	0.660	0.472	38.02
0.5	Mean	0.516	0.177	0.149	0.300	0.329	0.312	0.166	0.075	−0.141
	Var $\times 10^{2}$	4.305	3.602	0.689	1.529	3.657	6.121	0.572	0.454	33.05
	MSE $\times 10^{2}$	4.327 *	4.188	0.950	2.532	3.739	6.128	0.689	0.514	39.75
1	Mean	0.503	0.162	0.167	0.340	0.346	0.272	0.183	0.092	−0.194
	Var $\times 10^{2}$	4.432	3.750	0.989	2.266	3.911	6.436	0.772	0.726	47.97
	MSE $\times 10^{2}$	4.428	4.130 *	1.100	2.619	4.119	6.507	0.799	0.731	52.14

**Table 15:** Sample mean, variance, and MSE of estimators when $\theta ={\left(0.5,0.1,0.2,0.4,0.3,0.3,0.2,0.1,-0.4\right)}^{T}$, n=200, and (p,γ)=(0.03,10).

α		${\hat{\omega }}_{1}$	${\hat{a}}_{1}$	${\hat{b}}_{11}$	${\hat{b}}_{12}$	${\hat{\omega }}_{2}$	${\hat{a}}_{2}$	${\hat{b}}_{21}$	${\hat{b}}_{22}$	$\hat{\delta }$
0(CMLE)	Mean	0.736	0.312	0.084	0.165	0.497	0.416	0.103	0.047	0.291
	Var $\times 10^{2}$	16.70	8.406	0.635	1.549	12.16	10.20	0.674	0.401	33.37
	MSE $\times 10^{2}$	22.23	12.91	1.971	7.078	16.04	11.54	1.610	0.680	81.11
0.1	Mean	0.600	0.264	0.092	0.181	0.381	0.368	0.110	0.047	0.032
	Var $\times 10^{2}$	8.133	5.973	0.445	1.314	5.185	7.196	0.442	0.233	27.95
	MSE $\times 10^{2}$	9.120	8.669	1.613	6.104	5.843	7.657	1.255	0.515	46.63
0.2	Mean	0.570	0.252	0.095	0.188	0.367	0.353	0.110	0.050	−0.012
	Var $\times 10^{2}$	7.112	5.817	0.451	1.441	4.576	6.859	0.446	0.244	28.91
	MSE $\times 10^{2}$	7.592	8.112	1.544	5.920	5.016	7.136	1.250	0.495 *	43.96
0.3	Mean	0.563	0.235	0.100	0.200	0.366	0.338	0.113	0.054	−0.046
	Var $\times 10^{2}$	6.572	5.489	0.513	1.736	4.477	6.853	0.502	0.294	29.07
	MSE $\times 10^{2}$	6.965	7.299	1.513	5.748	4.910	6.988	1.263	0.503	41.58
0.5	Mean	0.553	0.193	0.113	0.235	0.369	0.294	0.124	0.066	−0.101
	Var $\times 10^{2}$	6.273	4.985	0.736	2.594	4.586	7.030	0.689	0.441	29.86
	MSE $\times 10^{2}$	6.548	5.840	1.484	5.318	5.061	7.027	1.263	0.555	38.79 *
1	Mean	0.552	0.126	0.166	0.340	0.383	0.227	0.176	0.104	−0.262
	Var $\times 10^{2}$	4.097	3.239	1.193	3.141	3.787	6.138	1.084	0.816	46.47
	MSE $\times 10^{2}$	4.360 *	3.304 *	1.307 *	3.502 *	4.479 *	6.658 *	1.141 *	0.817	48.32

**Table 16:** Sample mean, variance, and MSE of estimators when $\theta ={\left(0.5,0.1,0.2,0.4,0.3,0.3,0.2,0.1,-0.4\right)}^{T}$, n=200, and (p,γ)=(0.05,10).

α		${\hat{\omega }}_{1}$	${\hat{a}}_{1}$	${\hat{b}}_{11}$	${\hat{b}}_{12}$	${\hat{\omega }}_{2}$	${\hat{a}}_{2}$	${\hat{b}}_{21}$	${\hat{b}}_{22}$	$\hat{\delta }$
0(CMLE)	Mean	0.829	0.378	0.064	0.102	0.613	0.435	0.081	0.037	0.749
	Var $\times 10^{2}$	27.18	10.13	0.521	0.889	18.60	10.72	0.611	0.307	39.05
	MSE $\times 10^{2}$	37.97	17.82	2.375	9.795	28.35	12.52	2.018	0.702	171.0
0.1	Mean	0.652	0.313	0.070	0.114	0.441	0.374	0.082	0.034	0.172
	Var $\times 10^{2}$	12.51	7.707	0.348	0.815	7.550	8.532	0.365	0.162	29.09
	MSE $\times 10^{2}$	14.81	12.21	2.040	9.010	9.521	9.078	1.748	0.592	61.80
0.2	Mean	0.612	0.294	0.071	0.117	0.417	0.354	0.080	0.035	0.097
	Var $\times 10^{2}$	9.876	7.005	0.317	0.871	6.395	8.221	0.332	0.147	30.13
	MSE $\times 10^{2}$	11.13	10.74	1.979	8.885	7.751	8.510	1.768	0.571	54.75
0.3	Mean	0.604	0.283	0.073	0.121	0.414	0.343	0.081	0.037	0.063
	Var $\times 10^{2}$	9.469	7.048	0.348	1.031	6.125	8.167	0.358	0.167	30.74
	MSE $\times 10^{2}$	10.54	10.38	1.970	8.819	7.414	8.347	1.771	0.567 *	52.11
0.5	Mean	0.607	0.241	0.085	0.151	0.420	0.310	0.091	0.048	−0.006
	Var $\times 10^{2}$	8.559	6.604	0.565	1.957	5.915	8.036	0.536	0.337	32.13
	MSE $\times 10^{2}$	9.688	8.590	1.881	8.142	7.350	8.038	1.713	0.608	47.63 *
1	Mean	0.600	0.135	0.146	0.292	0.425	0.220	0.152	0.097	−0.195
	Var $\times 10^{2}$	5.457	4.147	1.395	4.172	4.697	6.676	1.279	0.911	46.82
	MSE $\times 10^{2}$	6.453 *	4.268 *	1.690 *	5.343 *	6.263 *	7.302 *	1.508 *	0.910	50.98

**Table 17:** Parameter estimates for bivariate Poisson integer-valued generalized autoregressive conditional heteroscedastic (INGARCH) model for crime data; the symbol ${}^{•}$ represents the minimal $\hat{\mathrm{AMSE}}$.

α	${\hat{\omega }}_{1}$	${\hat{a}}_{1}$	${\hat{b}}_{11}$	${\hat{b}}_{12}$	${\hat{\omega }}_{2}$	${\hat{a}}_{2}$	${\hat{b}}_{21}$	${\hat{b}}_{22}$	$\hat{\delta }$	$\hat{\mathrm{AMSE}}$
0(CMLE)	0.019	0.779	0.125	0.073	0.032	0.865	0.090	0.057	1.312	1.578
	(0.054)	(0.290)	(0.166)	(0.075)	(0.028)	(0.090)	(0.032)	(0.086)	(1.096)	
0.1	0.034	0.609	0.172	0.094	0.097	0.654	0.095	0.156	0.685	0.699 ${}^{•}$
	(0.034)	(0.149)	(0.104)	(0.026)	(0.047)	(0.091)	(0.043)	(0.069)	(0.678)	
0.2	0.026	0.643	0.134	0.087	0.117	0.575	0.124	0.159	0.509	0.858
	(0.032)	(0.163)	(0.109)	(0.026)	(0.060)	(0.121)	(0.052)	(0.069)	(0.692)	
0.3	0.021	0.666	0.113	0.085	0.129	0.523	0.154	0.155	0.401	0.991
	(0.029)	(0.149)	(0.096)	(0.027)	(0.067)	(0.133)	(0.053)	(0.068)	(0.710)	
0.4	0.019	0.673	0.107	0.085	0.130	0.508	0.176	0.145	0.356	1.081
	(0.029)	(0.143)	(0.093)	(0.029)	(0.067)	(0.135)	(0.055)	(0.069)	(0.736)	
0.5	0.018	0.675	0.105	0.086	0.125	0.514	0.196	0.131	0.365	1.108
	(0.029)	(0.138)	(0.093)	(0.032)	(0.065)	(0.136)	(0.059)	(0.071)	(0.768)	
0.6	0.017	0.676	0.104	0.088	0.119	0.527	0.216	0.115	0.418	1.094
	(0.029)	(0.133)	(0.091)	(0.036)	(0.062)	(0.135)	(0.065)	(0.073)	(0.807)	
0.7	0.017	0.675	0.104	0.089	0.114	0.540	0.238	0.100	0.509	1.073
	(0.029)	(0.130)	(0.092)	(0.041)	(0.059)	(0.133)	(0.075)	(0.075)	(0.859)	
0.8	0.018	0.674	0.104	0.090	0.111	0.551	0.261	0.087	0.638	1.079
	(0.031)	(0.130)	(0.094)	(0.045)	(0.057)	(0.133)	(0.089)	(0.076)	(0.929)	
0.9	0.018	0.672	0.104	0.091	0.109	0.560	0.285	0.076	0.808	1.158
	(0.033)	(0.133)	(0.098)	(0.050)	(0.056)	(0.134)	(0.105)	(0.077)	(1.021)	
1	0.019	0.668	0.104	0.092	0.108	0.568	0.312	0.066	1.025	1.383
	(0.035)	(0.138)	(0.103)	(0.054)	(0.057)	(0.136)	(0.122)	(0.079)	(1.143)	

**Table 18:** Parameter estimates for bivariate Poisson INGARCH model for cleaned data; the symbol ${}^{•}$ represents the minimal $\hat{\mathrm{AMSE}}$.

α	${\hat{\omega }}_{1}$	${\hat{a}}_{1}$	${\hat{b}}_{11}$	${\hat{b}}_{12}$	${\hat{\omega }}_{2}$	${\hat{a}}_{2}$	${\hat{b}}_{21}$	${\hat{b}}_{22}$	$\hat{\delta }$	$\hat{\mathrm{AMSE}}$
0(CMLE)	0.0018	0.943	0.025	0.021	0.092	0.682	0.067	0.184	0.118	0.430 ${}^{•}$
	(0.007)	(0.118)	(0.084)	(0.017)	(0.050)	(0.115)	(0.069)	(0.069)	(0.609)	
0.1	0.0002	0.942	0.026	0.022	0.074	0.680	0.066	0.183	0.159	0.445
	(0.006)	(0.097)	(0.076)	(0.013)	(0.035)	(0.084)	(0.063)	(0.053)	(0.626)	
0.2	0.0001	0.940	0.025	0.023	0.066	0.679	0.066	0.182	0.199	0.497
	(0.009)	(0.100)	(0.079)	(0.011)	(0.032)	(0.075)	(0.063)	(0.049)	(0.657)	
0.3	0.0001	0.939	0.024	0.023	0.060	0.678	0.066	0.182	0.220	0.549
	(0.010)	(0.102)	(0.082)	(0.011)	(0.032)	(0.073)	(0.064)	(0.049)	(0.688)	
0.4	0.0001	0.939	0.024	0.023	0.057	0.678	0.066	0.182	0.228	0.591
	(0.010)	(0.104)	(0.086)	(0.011)	(0.032)	(0.074)	(0.066)	(0.049)	(0.715)	
0.5	0.0001	0.938	0.025	0.023	0.056	0.676	0.066	0.182	0.293	0.665
	(0.010)	(0.104)	(0.087)	(0.011)	(0.034)	(0.076)	(0.068)	(0.051)	(0.742)	
0.6	0.0001	0.938	0.024	0.023	0.054	0.677	0.066	0.182	0.263	0.688
	(0.009)	(0.102)	(0.088)	(0.011)	(0.035)	(0.077)	(0.071)	(0.053)	(0.769)	
0.7	0.0002	0.939	0.024	0.023	0.051	0.678	0.066	0.182	0.237	0.719
	(0.008)	(0.106)	(0.093)	(0.012)	(0.035)	(0.078)	(0.074)	(0.055)	(0.795)	
0.8	0.0002	0.940	0.015	0.027	0.053	0.678	0.067	0.179	0.100	0.812
	(0.011)	(0.126)	(0.093)	(0.013)	(0.038)	(0.081)	(0.076)	(0.057)	(0.873)	
0.9	0.0001	0.944	0.011	0.028	0.050	0.679	0.068	0.176	−0.029	0.924
	(0.012)	(0.150)	(0.108)	(0.015)	(0.039)	(0.083)	(0.080)	(0.058)	(0.933)	
1	0.0002	0.944	0.010	0.028	0.054	0.677	0.070	0.173	0.010	1.079
	(0.012)	(0.151)	(0.107)	(0.015)	(0.044)	(0.087)	(0.084)	(0.061)	(1.012)	

**Table 19:** Parameter estimates for bivariate Poisson INGARCH model for artificial data; the symbol ${}^{•}$ represents the minimal $\hat{\mathrm{AMSE}}$.

α	${\hat{\omega }}_{1}$	${\hat{a}}_{1}$	${\hat{b}}_{11}$	${\hat{b}}_{12}$	${\hat{\omega }}_{2}$	${\hat{a}}_{2}$	${\hat{b}}_{21}$	${\hat{b}}_{22}$	$\hat{\delta }$	$\hat{\mathrm{AMSE}}$
0(CMLE)	1.507	0.274	0.438	0.000	0.976	0.410	0.000	0.375	0.772	9.468
	(0.442)	(0.102)	(0.053)	(0.031)	(0.241)	(0.069)	(0.029)	(0.048)	(2.939)	
0.1	1.442	0.274	0.449	0.000	0.952	0.412	0.000	0.372	0.308	7.647
	(0.432)	(0.100)	(0.053)	(0.031)	(0.236)	(0.066)	(0.030)	(0.048)	(2.688)	
0.2	1.402	0.273	0.457	0.000	0.918	0.417	0.000	0.371	−0.064	6.611
	(0.443)	(0.102)	(0.054)	(0.033)	(0.237)	(0.065)	(0.031)	(0.049)	(2.487)	
0.3	1.373	0.271	0.464	0.000	0.883	0.422	0.000	0.372	−0.329	6.216 ${}^{•}$
	(0.465)	(0.105)	(0.056)	(0.034)	(0.242)	(0.065)	(0.033)	(0.050)	(2.367)	
0.4	1.349	0.269	0.471	0.000	0.849	0.425	0.000	0.375	−0.485	6.276
	(0.494)	(0.111)	(0.058)	(0.036)	(0.250)	(0.064)	(0.034)	(0.052)	(2.339)	
0.5	1.326	0.268	0.476	0.000	0.817	0.427	0.000	0.380	−0.540	6.607
	(0.528)	(0.118)	(0.060)	(0.039)	(0.259)	(0.064)	(0.037)	(0.054)	(2.388)	
0.6	1.302	0.267	0.482	0.000	0.786	0.428	0.000	0.386	−0.509	7.031
	(0.567)	(0.126)	(0.063)	(0.041)	(0.271)	(0.064)	(0.039)	(0.056)	(2.476)	
0.7	1.277	0.267	0.487	0.000	0.758	0.428	0.000	0.394	−0.407	7.412
	(0.610)	(0.135)	(0.066)	(0.044)	(0.285)	(0.064)	(0.042)	(0.058)	(2.566)	
0.8	1.250	0.267	0.491	0.000	0.732	0.427	0.000	0.401	−0.250	7.698
	(0.657)	(0.145)	(0.069)	(0.047)	(0.299)	(0.064)	(0.045)	(0.060)	(2.639)	
0.9	1.223	0.267	0.496	0.000	0.708	0.425	0.000	0.410	−0.055	7.916
	(0.707)	(0.156)	(0.072)	(0.050)	(0.314)	(0.065)	(0.048)	(0.062)	(2.688)	
1	1.196	0.268	0.500	0.000	0.686	0.423	0.000	0.418	0.165	8.131
	(0.761)	(0.168)	(0.076)	(0.053)	(0.330)	(0.065)	(0.051)	(0.064)	(2.719)	

## Data Availability

Publicly available datasets were analyzed in this study. This data can be found here: data.gov.au (accessed on 19 March 2021).

## Appendix A

In this Appendix, we provide the proofs for Theorems 1 and 2 in Section 3 when α>0. We refer to Cui and Zhu [20] for the case of α=0. In what follows, we denote *V* and ρ∈(0,1) as a generic positive integrable random variable and a generic constant, respectively, and $H_{\alpha ,n}\left(\theta \right)=n^{-1}\sum_{t=1}^{n}h_{\alpha ,t}\left(\theta \right)$. Furthermore, we employ the notation $\lambda_{t}=\lambda_{t}\left(\theta \right)$, ${\tilde{\lambda }}_{t}={\tilde{\lambda }}_{t}\left(\theta \right)$, $\lambda_{t}^{0}=\lambda_{t}\left(\theta^{0}\right)$, $\lambda_{t,i}=\lambda_{t,i}\left(\theta_{i}\right)$, ${\tilde{\lambda }}_{t,i}={\tilde{\lambda }}_{t,i}\left(\theta_{i}\right)$, $\lambda_{t,i}^{0}=\lambda_{t,i}\left(\theta_{i}^{0}\right)$ for i=1,2, and $u\left(y,\lambda \right)=e^{-y}-e^{-c\lambda }$ for brevity.

**Lemma** **A1.**
*Under* ***(A1)*** *–* ***(A3)*** *, we have for i=1,2,*

(i) *$E(\sup_{\theta_{i}\in \Theta_{i}}\lambda_{t,i})<\infty$.*
(ii) *$\lambda_{t,i}=\lambda_{t,i}^{0}$ a.s. implies $\theta_{i}=\theta_{i}^{0}$.*
(iii) *$\lambda_{t,i}$ is twice continuously differentiable with respect to $\theta_{i}$ and satisfies*
  $$\begin{array}{c} E{\left(\underset{\theta_{i}\in \Theta_{i}}{\sup}{∥\frac{\partial \lambda_{t,i}}{\partial \theta_{i}}∥}_{1}\right)}^{4}<\infty \;and\;E{\left(\underset{\theta_{i}\in \Theta_{i}}{\sup}{∥\frac{\partial^{2}\lambda_{t,i}}{\partial \theta_{i}\partial \theta_{i}^{T}}∥}_{1}\right)}^{2}<\infty . \end{array}$$
(iv) *$\sup_{\theta_{i}\in \Theta_{i}}{∥\frac{\partial \lambda_{t,i}}{\partial \theta_{i}}-\frac{\partial {\tilde{\lambda }}_{t,i}}{\partial \theta_{i}}∥}_{1}\leq V\rho^{t}\;a.s.$*
(v) *$\nu^{T}\frac{\partial \lambda_{t,i}^{0}}{\partial \theta_{i}}=0$ a.s. implies ν=0.*
(vi) *$\sup_{\theta_{i}\in \Theta_{i}}\left|\lambda_{t,i}-{\tilde{\lambda }}_{t,i}\right|\leq V\rho^{t}$ a.s.*

**Proof.** By recursion of (2), we have

$$\begin{array}{ccc} \lambda_{t} & = & {\left(I_{2}-A\right)}^{-1}\omega +\sum_{k=0}^{\infty }A^{k}BY_{t-k-1},\\ {\tilde{\lambda }}_{t} & = & \left(I_{2}+A+\cdots +A^{t-2}\right)\omega +A^{t-1}{\tilde{\lambda }}_{1}+\sum_{k=0}^{t-2}A^{k}BY_{t-k-1} \end{array}$$

and thus, for i=1,2,

$$\begin{array}{ccc} \lambda_{t,i} & = & \frac{\omega_{i}}{1-a_{i}}+\sum_{k=0}^{\infty }a_{i}^{k}\left(b_{i1}Y_{t-k-1,1}+b_{i2}Y_{t-k-1,2}\right),\\ {\tilde{\lambda }}_{t,i} & = & \frac{\omega_{i}}{1-a_{i}}+\sum_{k=0}^{t-2}a_{i}^{k}\left(b_{i1}Y_{t-k-1,1}+b_{i2}Y_{t-k-1,2}\right), \end{array}$$

where $I_{2}$ denotes 2×2 identity matrix and the initial value ${\tilde{\lambda }}_{1,i}$ is taken as $\omega_{i}/\left(1-a_{i}\right)$ for simplicity. Subsequently, (i)−(v) can be shown following the arguments in the proof of Theorem 3 in Kang and Lee [44]. For (vi), let $\rho =\sup_{\theta_{i}\in \Theta_{i}}a_{i}<1$. Afterwards, from (2), it holds that

$$\begin{array}{c} \underset{\theta_{i}\in \Theta_{i}}{\sup}|\lambda_{t,i}-{\tilde{\lambda }}_{t,i}|=\underset{\theta_{i}\in \Theta_{i}}{\sup}|a_{i}\left(\lambda_{t-1,i}-{\tilde{\lambda }}_{t-1,i}\right)|=\cdots =\underset{\theta_{i}\in \Theta_{i}}{\sup}\left|a_{i}^{t-1}\left(\lambda_{1,i}-{\tilde{\lambda }}_{1,i}\right)\right|\leq V\rho^{t} \end{array}$$

with $V=\sup_{\theta_{i}\in \Theta_{i}}\left|\lambda_{1,i}-{\tilde{\lambda }}_{1,i}\right|/\rho$. Therefore, the lemma is established. □

**Lemma** **A2.**
*Under* ***(A1)*** *–* ***(A3)*** *, we have*

$$\begin{array}{c} \underset{\theta \in \Theta }{\sup}\left|H_{\alpha ,n}\left(\theta \right)-{\tilde{H}}_{\alpha ,n}\left(\theta \right)\right|\overset{a.s.}{⟶}0\;\;as\;\;n\to \infty . \end{array}$$

**Proof.** It is sufficient to show that

$$\begin{array}{c} \underset{\theta \in \Theta }{\sup}\left|h_{\alpha ,t}\left(\theta \right)-{\tilde{h}}_{\alpha ,t}\left(\theta \right)\right|\overset{a.s.}{⟶}0\;\;as\;\;t\to \infty . \end{array}$$

We write

$$\begin{array}{ccc} & & |h_{\alpha ,t}\left(\theta \right)-{\tilde{h}}_{\alpha ,t}\left(\theta \right)|\\ & \leq & \left|\sum_{y_{1}=0}^{\infty }\sum_{y_{2}=0}^{\infty }\left\{f_{\theta }^{1+\alpha }\left(y|\lambda_{t}\right)-f_{\theta }^{1+\alpha }\left(y|{\tilde{\lambda }}_{t}\right)\right\}\right|+\left(1+\frac{1}{\alpha }\right)\left|f_{\theta }^{\alpha }\left(Y_{t}|\lambda_{t}\right)-f_{\theta }^{\alpha }\left(Y_{t}|{\tilde{\lambda }}_{t}\right)\right|\\ & := & I_{t}\left(\theta \right)+II_{t}\left(\theta \right). \end{array}$$

From (A1), (A2), and the mean value theorem (MVT), we have

$$\begin{array}{ccc} I_{t}\left(\theta \right) & = & \left(1+\alpha \right)\left|\sum_{y_{1}=0}^{\infty }\sum_{y_{2}=0}^{\infty }f_{\theta }^{1+\alpha }\left(y|\lambda_{t}^{*}\right)\left\{\frac{y_{1}}{\lambda_{t,1}^{*}}-1+\frac{c\delta e^{-c\lambda_{t,1}^{*}}u\left(y_{2},\lambda_{t,2}^{*}\right)}{\varphi (y,\lambda_{t}^{*},\delta )}\right\}\left(\lambda_{t,1}-{\tilde{\lambda }}_{t,1}\right)\right.\\ & & \left.+\sum_{y_{1}=0}^{\infty }\sum_{y_{2}=0}^{\infty }f_{\theta }^{1+\alpha }\left(y|\lambda_{t}^{*}\right)\left\{\frac{y_{2}}{\lambda_{t,2}^{*}}-1+\frac{c\delta e^{-c\lambda_{t,2}^{*}}u\left(y_{1},\lambda_{t,1}^{*}\right)}{\varphi (y,\lambda_{t}^{*},\delta )}\right\}\left(\lambda_{t,2}-{\tilde{\lambda }}_{t,2}\right)\right|\\ & \leq & \left(1+\alpha \right)\left[\sum_{y_{1}=0}^{\infty }\sum_{y_{2}=0}^{\infty }f_{\theta }\left(y|\lambda_{t}^{*}\right)\left\{\frac{y_{1}}{\lambda_{t,1}^{*}}+1+\frac{c|\delta |e^{-c\lambda_{t,1}^{*}}\left|u\left(y_{2},\lambda_{t,2}^{*}\right)\right|}{\varphi (y,\lambda_{t}^{*},\delta )}\right\}\left|\lambda_{t,1}-{\tilde{\lambda }}_{t,1}\right|\right.\\ & & \left.+\sum_{y_{1}=0}^{\infty }\sum_{y_{2}=0}^{\infty }f_{\theta }\left(y|\lambda_{t}^{*}\right)\left\{\frac{y_{2}}{\lambda_{t,2}^{*}}+1+\frac{c|\delta |e^{-c\lambda_{t,2}^{*}}\left|u\left(y_{1},\lambda_{t,1}^{*}\right)\right|}{\varphi (y,\lambda_{t}^{*},\delta )}\right\}\left|\lambda_{t,2}-{\tilde{\lambda }}_{t,2}\right|\right]\\ & \leq & \left(1+\alpha \right)\left\{\left(1+1+\frac{2c\delta_{U}}{\varphi_{L}}\right)|\lambda_{t,1}-{\tilde{\lambda }}_{t,1}|+\left(1+1+\frac{2c\delta_{U}}{\varphi_{L}}\right)\left|\lambda_{t,2}-{\tilde{\lambda }}_{t,2}\right|\right\}\\ & = & 2\left(1+\alpha \right)\left(1+\frac{c\delta_{U}}{\varphi_{L}}\right)\left(\right|\lambda_{t,1}-{\tilde{\lambda }}_{t,1}\left|+\right|\lambda_{t,2}-{\tilde{\lambda }}_{t,2}\left|\right), \end{array}$$

where $\lambda_{t}^{*}={\left(\lambda_{t,1}^{*},\lambda_{t,2}^{*}\right)}^{T}$ and $\lambda_{t,i}^{*}$ is an intermediate point between $\lambda_{t,i}$ and ${\tilde{\lambda }}_{t,i}$ for i=1,2. Hence, $\sup_{\theta \in \Theta }I_{t}\left(\theta \right)$ converges to 0 a.s. as t→∞ by (vi) of Lemma A1. Because $\lambda_{t,i}^{*}=m\lambda_{t,i}+\left(1-m\right){\tilde{\lambda }}_{t,i}$ for some m∈(0,1), it holds that ${\left(\lambda_{t,i}^{*}\right)}^{-1}\leq {\left(m\lambda_{t,i}\right)}^{-1}\leq {\left(m\omega_{L}\right)}^{-1}$. Thus, we obtain

$$\begin{array}{ccc} II_{t}\left(\theta \right) & = & \left(1+\alpha \right)\left|f_{\theta }^{\alpha }\left(Y_{t}|\lambda_{t}^{*}\right)\left\{\frac{Y_{t,1}}{\lambda_{t,1}^{*}}-1+\frac{c\delta e^{-c\lambda_{t,1}^{*}}u\left(Y_{t,2},\lambda_{t,2}^{*}\right)}{\varphi (Y_{t},\lambda_{t}^{*},\delta )}\right\}\left(\lambda_{t,1}-{\tilde{\lambda }}_{t,1}\right)\right.\\ & & \left.+f_{\theta }^{\alpha }\left(Y_{t}|\lambda_{t}^{*}\right)\left\{\frac{Y_{t,2}}{\lambda_{t,2}^{*}}-1+\frac{c\delta e^{-c\lambda_{t,2}^{*}}u\left(Y_{t,1},\lambda_{t,1}^{*}\right)}{\varphi (Y_{t},\lambda_{t}^{*},\delta )}\right\}\left(\lambda_{t,2}-{\tilde{\lambda }}_{t,2}\right)\right|\\ & \leq & \left(1+\alpha \right)\left\{\left(\frac{Y_{t,1}}{m\omega_{L}}+1+\frac{2c\delta_{U}}{\varphi_{L}}\right)|\lambda_{t,1}-{\tilde{\lambda }}_{t,1}|+\left(\frac{Y_{t,2}}{m\omega_{L}}+1+\frac{2c\delta_{U}}{\varphi_{L}}\right)\left|\lambda_{t,2}-{\tilde{\lambda }}_{t,2}\right|\right\}. \end{array}$$

According to Lemma 2.1 in Straumann and Mikosch [45], together with (vi) of Lemma A1, $\sup_{\theta \in \Theta }II_{t}\left(\theta \right)$ converges to 0 a.s. as t→∞. Therefore, the lemma is verified. □

**Lemma** **A3.**
*Under* ***(A1)*** *–* ***(A3)*** *, we have*

$$\begin{array}{c} E\left(\underset{\theta \in \Theta }{\sup}\left|h_{\alpha ,t}\left(\theta \right)\right|\right)<\infty \;and\;if\;\theta \neq \theta^{0},\;then\;E\left(h_{\alpha ,t}\left(\theta \right)\right)>E\left(h_{\alpha ,t}\left(\theta^{0}\right)\right). \end{array}$$

**Proof.** Because

$$\begin{array}{c} |h_{\alpha ,t}\left(\theta \right)|\leq \sum_{y_{1}=0}^{\infty }\sum_{y_{2}=0}^{\infty }f_{\theta }^{1+\alpha }\left(y|\lambda_{t}\right)+\left(1+\frac{1}{\alpha }\right)f_{\theta }^{\alpha }\left(Y_{t}|\lambda_{t}\right)\leq 2+\frac{1}{\alpha }, \end{array}$$

the first part of the lemma is validated. Note that

$$\begin{array}{ccc} & & E\left(h_{\alpha ,t}\left(\theta \right)\right)-E\left(h_{\alpha ,t}\left(\theta^{0}\right)\right)\\ & = & E\left[E\left\{h_{\alpha ,t}\left(\theta \right)-h_{\alpha ,t}\left(\theta^{0}\right)|F_{t-1}\right\}\right]\\ & = & E\left[\sum_{y_{1}=0}^{\infty }\sum_{y_{2}=0}^{\infty }\left\{f_{\theta }^{1+\alpha }\left(y|\lambda_{t}\right)-\left(1+\frac{1}{\alpha }\right)f_{\theta }^{\alpha }\left(y|\lambda_{t}\right)f_{\theta^{0}}\left(y|\lambda_{t}\right)+\frac{1}{\alpha }f_{\theta^{0}}^{1+\alpha }\left(y|\lambda_{t}\right)\right\}\right]\\ & \geq & 0, \end{array}$$

where the equality holds if and only if $\delta =\delta^{0}$ and $\lambda_{t}=\lambda_{t}^{0}$ a.s. Therefore, the second part of the lemma is established by (ii) of Lemma A1. □

**Proof of Theorem 1.** We can write

$$\begin{array}{ccc} & & \underset{\theta \in \Theta }{\sup}\left|\frac{1}{n}\sum_{t=1}^{n}{\tilde{h}}_{\alpha ,t}\left(\theta \right)-E\left(h_{\alpha ,t}\left(\theta \right)\right)\right|\\ & \leq & \underset{\theta \in \Theta }{\sup}\left|\frac{1}{n}\sum_{t=1}^{n}{\tilde{h}}_{\alpha ,t}\left(\theta \right)-\frac{1}{n}\sum_{t=1}^{n}h_{\alpha ,t}\left(\theta \right)\right|+\underset{\theta \in \Theta }{\sup}\left|\frac{1}{n}\sum_{t=1}^{n}h_{\alpha ,t}\left(\theta \right)-E\left(h_{\alpha ,t}\left(\theta \right)\right)\right|. \end{array}$$

The first term on the RHS of the inequality converges to 0 a.s. from Lemma A2. Moreover, because $h_{\alpha ,t}\left(\theta \right)$ is stationary and ergodic with $E(\sup_{\theta \in \Theta }|h_{\alpha ,t}\left(\theta \right)|)<\infty$ by Lemma A3, the second term also converges to 0 a.s. Finally, as $E(h_{\alpha ,t}\left(\theta \right))$ has a unique minimum at $\theta^{0}$ from Lemma A3, the theorem is established. □

Now, we derive the first and second derivatives of $h_{\alpha ,t}\left(\theta \right)$. The first derivatives are obtained as

$$\begin{array}{ccc} \frac{\partial h_{\alpha ,t}\left(\theta \right)}{\partial \theta } & = & \left(1+\alpha \right){\left(D_{t,1}\left(\theta \right)s_{t,1}{\left(\theta_{1}\right)}^{T},D_{t,2}\left(\theta \right)s_{t,2}{\left(\theta_{2}\right)}^{T},D_{t,3}\left(\theta \right)\right)}^{T}\\ & = & \left(1+\alpha \right)\left(\begin{array}{ccc} D_{t,1}\left(\theta \right)I_{4} & 0_{4\times 4} & 0_{4\times 1}\\ 0_{4\times 4} & D_{t,2}\left(\theta \right)I_{4} & 0_{4\times 1}\\ 0_{1\times 4} & 0_{1\times 4} & D_{t,3}\left(\theta \right) \end{array}\right)\left(\begin{array}{c} s_{t,1}\left(\theta_{1}\right)\\ s_{t,2}\left(\theta_{2}\right)\\ 1 \end{array}\right)\\ & := & \left(1+\alpha \right)D_{t}\left(\theta \right)\Lambda_{t}\left(\theta \right), \end{array}$$

where $I_{4}$ denotes the 4×4 identity matrix, $0_{m\times n}$ means the m×n matrix with zero elements, and

$$\begin{array}{ccc} s_{t,i}\left(\theta_{i}\right) & = & \frac{\partial \lambda_{t,i}}{\partial \theta_{i}}\;\;\mathrm{for}\;\;i=1,2,\\ D_{t,i}\left(\theta \right) & = & \sum_{y_{1}=0}^{\infty }\sum_{y_{2}=0}^{\infty }f_{\theta }^{1+\alpha }\left(y|\lambda_{t}\right)\left\{\frac{y_{i}}{\lambda_{t,i}}-1+\frac{c\delta e^{-c\lambda_{t,i}}u\left(y_{j},\lambda_{t,j}\right)}{\varphi (y,\lambda_{t},\delta )}\right\}\\ & & -f_{\theta }^{\alpha }\left(Y_{t}|\lambda_{t}\right)\left\{\frac{Y_{t,i}}{\lambda_{t,i}}-1+\frac{c\delta e^{-c\lambda_{t,i}}u\left(Y_{t,j},\lambda_{t,j}\right)}{\varphi (Y_{t},\lambda_{t},\delta )}\right\}\;\;\mathrm{for}\;\;\left(i,j\right)=\left(1,2\right),\left(2,1\right),\\ D_{t,3}\left(\theta \right) & = & \sum_{y_{1}=0}^{\infty }\sum_{y_{2}=0}^{\infty }f_{\theta }^{1+\alpha }\left(y|\lambda_{t}\right)\frac{u\left(y_{1},\lambda_{t,1}\right)u\left(y_{2},\lambda_{t,2}\right)}{\varphi (y,\lambda_{t},\delta )}-f_{\theta }^{\alpha }\left(Y_{t}|\lambda_{t}\right)\frac{u\left(Y_{t,1},\lambda_{t,1}\right)u\left(Y_{t,2},\lambda_{t,2}\right)}{\varphi (Y_{t},\lambda_{t},\delta )}. \end{array}$$

The second derivatives are expressed as

$$\begin{array}{ccc} \frac{\partial^{2}h_{\alpha ,t}\left(\theta \right)}{\partial \theta \partial \theta^{T}} & = & \left(1+\alpha \right)\left(\begin{array}{ccc} F_{t,11}\left(\theta \right)s_{t,1}\left(\theta_{1}\right)s_{t,1}{\left(\theta_{1}\right)}^{T} & F_{t,12}\left(\theta \right)s_{t,1}\left(\theta_{1}\right)s_{t,2}{\left(\theta_{2}\right)}^{T} & F_{t,13}\left(\theta \right)s_{t,1}\left(\theta_{1}\right)\\ F_{t,21}\left(\theta \right)s_{t,2}\left(\theta_{2}\right)s_{t,1}{\left(\theta_{1}\right)}^{T} & F_{t,22}\left(\theta \right)s_{t,2}\left(\theta_{2}\right)s_{t,2}{\left(\theta_{2}\right)}^{T} & F_{t,23}\left(\theta \right)s_{t,2}\left(\theta_{2}\right)\\ F_{t,31}\left(\theta \right)s_{t,1}{\left(\theta_{1}\right)}^{T} & F_{t,32}\left(\theta \right)s_{t,2}{\left(\theta_{2}\right)}^{T} & F_{t,33}\left(\theta \right) \end{array}\right)\\ & & +\left(1+\alpha \right)\left(\begin{array}{ccc} D_{t,1}\left(\theta \right)s_{t,11}\left(\theta_{1}\right) & 0_{4\times 4} & 0_{4\times 1}\\ 0_{4\times 4} & D_{t,2}\left(\theta \right)s_{t,22}\left(\theta_{2}\right) & 0_{4\times 1}\\ 0_{1\times 4} & 0_{1\times 4} & 0 \end{array}\right)\\ & := & \left(1+\alpha \right)\left\{F_{t}\left(\theta \right)+D_{t}\left(\theta \right)\frac{\partial \Lambda_{t}\left(\theta \right)}{\partial \theta^{T}}\right\}, \end{array}$$

where

$$\begin{array}{ccc} s_{t,ii}\left(\theta_{i}\right) & = & \frac{\partial^{2}\lambda_{t,i}}{\partial \theta_{i}\partial \theta_{i}^{T}}\;\;\mathrm{for}\;\;i=1,2,\\ F_{t,ii}\left(\theta \right) & = & \sum_{y_{1}=0}^{\infty }\sum_{y_{2}=0}^{\infty }f_{\theta }^{1+\alpha }\left(y|\lambda_{t}\right)\left[\left(1+\alpha \right){\left\{\frac{y_{i}}{\lambda_{t,i}}-1+\frac{c\delta e^{-c\lambda_{t,i}}u\left(y_{j},\lambda_{t,j}\right)}{\varphi (y,\lambda_{t},\delta )}\right\}}^{2}\right.\\ & & \;\;\;\;\;\left.-\frac{y_{i}}{\lambda_{t,i}^{2}}-\frac{c^{2}\delta e^{-c\lambda_{t,i}}u\left(y_{j},\lambda_{t,j}\right)\left\{1+\delta e^{-y_{i}}u\left(y_{j},\lambda_{t,j}\right)\right\}}{\varphi {\left(y,\lambda_{t},\delta \right)}^{2}}\right]\\ & & -f_{\theta }^{\alpha }\left(Y_{t}|\lambda_{t}\right)\left[\alpha {\left\{\frac{Y_{t,i}}{\lambda_{t,i}}-1+\frac{c\delta e^{-c\lambda_{t,i}}u\left(Y_{t,j},\lambda_{t,j}\right)}{\varphi (Y_{t},\lambda_{t},\delta )}\right\}}^{2}\right.\\ & & \;\;\;\;\;\left.-\frac{Y_{t,i}}{\lambda_{t,i}^{2}}-\frac{c^{2}\delta e^{-c\lambda_{t,i}}u\left(Y_{t,j},\lambda_{t,j}\right)\left\{1+\delta e^{-Y_{t,i}}u\left(Y_{t,j},\lambda_{t,j}\right)\right\}}{\varphi {\left(Y_{t},\lambda_{t},\delta \right)}^{2}}\right]\\ & & \;\;\mathrm{for}\;\;(i,j)=(1,2),(2,1),\\ F_{t,33}\left(\theta \right) & = & \alpha \sum_{y_{1}=0}^{\infty }\sum_{y_{2}=0}^{\infty }f_{\theta }^{1+\alpha }\left(y|\lambda_{t}\right){\left\{\frac{u\left(y_{1},\lambda_{t,1}\right)u\left(y_{2},\lambda_{t,2}\right)}{\varphi (y,\lambda_{t},\delta )}\right\}}^{2}\\ & & -\left(\alpha -1\right)f_{\theta }^{\alpha }\left(Y_{t}|\lambda_{t}\right){\left\{\frac{u\left(Y_{t,1},\lambda_{t,1}\right)u\left(Y_{t,2},\lambda_{t,2}\right)}{\varphi (Y_{t},\lambda_{t},\delta )}\right\}}^{2},\\ F_{t,12}\left(\theta \right) & = & \sum_{y_{1}=0}^{\infty }\sum_{y_{2}=0}^{\infty }f_{\theta }^{1+\alpha }\left(y|\lambda_{t}\right)\left[\left(1+\alpha \right)\left\{\frac{y_{1}}{\lambda_{t,1}}-1+\frac{c\delta e^{-c\lambda_{t,1}}u\left(y_{2},\lambda_{t,2}\right)}{\varphi (y,\lambda_{t},\delta )}\right\}\right.\\ & & \;\;\;\;\;\;\;\;\;\;\left.\times \left\{\frac{y_{2}}{\lambda_{t,2}}-1+\frac{c\delta e^{-c\lambda_{t,2}}u\left(y_{1},\lambda_{t,1}\right)}{\varphi (y,\lambda_{t},\delta )}\right\}+\frac{c^{2}\delta e^{-c(\lambda_{t,1}+\lambda_{t,2})}}{\varphi {\left(y,\lambda_{t},\delta \right)}^{2}}\right]\\ & & -f_{\theta }^{\alpha }\left(Y_{t}|\lambda_{t}\right)\left[\alpha \left\{\frac{Y_{t,1}}{\lambda_{t,1}}-1+\frac{c\delta e^{-c\lambda_{t,1}}u\left(Y_{t,2},\lambda_{t,2}\right)}{\varphi (Y_{t},\lambda_{t},\delta )}\right\}\right.\\ & & \;\;\;\;\;\;\;\;\;\;\left.\times \left\{\frac{Y_{t,2}}{\lambda_{t,2}}-1+\frac{c\delta e^{-c\lambda_{t,2}}u\left(Y_{t,1},\lambda_{t,1}\right)}{\varphi (Y_{t},\lambda_{t},\delta )}\right\}+\frac{c^{2}\delta e^{-c(\lambda_{t,1}+\lambda_{t,2})}}{\varphi {\left(Y_{t},\lambda_{t},\delta \right)}^{2}}\right],\\ F_{t,i3}\left(\theta \right) & = & \sum_{y_{1}=0}^{\infty }\sum_{y_{2}=0}^{\infty }f_{\theta }^{1+\alpha }\left(y|\lambda_{t}\right)\left[\left(1+\alpha \right)\left\{\frac{y_{i}}{\lambda_{t,i}}-1+\frac{c\delta e^{-c\lambda_{t,i}}u\left(y_{j},\lambda_{t,j}\right)}{\varphi (y,\lambda_{t},\delta )}\right\}\right.\\ & & \;\;\;\;\;\;\;\;\;\;\;\;\left.\times \frac{u\left(y_{1},\lambda_{t,1}\right)u\left(y_{2},\lambda_{t,2}\right)}{\varphi (y,\lambda_{t},\delta )}+\frac{ce^{-c\lambda_{t,i}}u\left(y_{j},\lambda_{t,j}\right)}{\varphi {\left(y,\lambda_{t},\delta \right)}^{2}}\right]\\ & & -f_{\theta }^{\alpha }\left(Y_{t}|\lambda_{t}\right)\left[\alpha \left\{\frac{Y_{t,i}}{\lambda_{t,i}}-1+\frac{c\delta e^{-c\lambda_{t,i}}u\left(Y_{t,j},\lambda_{t,j}\right)}{\varphi (Y_{t},\lambda_{t},\delta )}\right\}\frac{u\left(Y_{t,1},\lambda_{t,1}\right)u\left(Y_{t,2},\lambda_{t,2}\right)}{\varphi (Y_{t},\lambda_{t},\delta )}\right.\\ & & \;\;\;\;\;\;\;\;\;\;\;\;\left.+\frac{ce^{-c\lambda_{t,i}}u\left(Y_{t,j},\lambda_{t,j}\right)}{\varphi {\left(Y_{t},\lambda_{t},\delta \right)}^{2}}\right]\;\;\mathrm{for}\;\;\left(i,j\right)=\left(1,2\right),\left(2,1\right). \end{array}$$

The following four lemmas are helpful for proving Theorem 2.

**Lemma** **A4.**
*Let ${\tilde{D}}_{t,i}\left(\theta \right)$ denote the counterpart of $D_{t,i}\left(\theta \right)$ by substituting $\lambda_{t}$ with ${\tilde{\lambda }}_{t}$ for i=1,2,3. Subsequently, under* ***(A1)*** *–* ***(A3)*** *, we have that for i=1,2,*

$$\begin{array}{ccc} & & |D_{t,i}\left(\theta \right)|\leq C\left(Y_{t,i}+1\right),\;\;|{\tilde{D}}_{t,i}\left(\theta \right)|\leq C\left(Y_{t,i}+1\right),\;\;|D_{t,3}\left(\theta \right)\left|\leq C,\;\;\right|{\tilde{D}}_{t,3}\left(\theta \right)|\leq C,\\ & & |F_{t,ii}\left(\theta \right)|\leq C\left(Y_{t,i}^{2}+Y_{t,i}+1\right),\;\;|F_{t,33}\left(\theta \right)\left|\leq C,\;\;\right|F_{t,12}\left(\theta \right)|\leq C\left(Y_{t,1}Y_{t,2}+Y_{t,1}+Y_{t,2}+1\right),\\ & & |F_{t,i3}\left(\theta \right)|\leq C\left(Y_{t,i}+1\right), \end{array}$$

*and for (i,j)=(1,2),(2,1),*

$$\begin{array}{cc} |D_{t,i}\left(\theta \right)-{\tilde{D}}_{t,i}\left(\theta \right)| & \leq C\left(Y_{t,i}^{2}+Y_{t,i}+1\right)\left|\lambda_{t,i}-{\tilde{\lambda }}_{t,i}\right|\\ & +C\left(Y_{t,1}Y_{t,2}+Y_{t,1}+Y_{t,2}+1\right)\left|\lambda_{t,j}-{\tilde{\lambda }}_{t,j}\right|,\\ |D_{t,3}\left(\theta \right)-{\tilde{D}}_{t,3}\left(\theta \right)|\leq & C\left(Y_{t,1}+1\right)|\lambda_{t,1}-{\tilde{\lambda }}_{t,1}|+C\left(Y_{t,2}+1\right)\left|\lambda_{t,2}-{\tilde{\lambda }}_{t,2}\right|, \end{array}$$

*where C is some positive constant.*

**Proof.** From **(A1)**–**(A3)** and the fact that $\lambda_{t,i}^{-1}\leq \omega_{L}^{-1}$, we obtain

$$\begin{array}{ccc} |D_{t,i}\left(\theta \right)| & \leq & \sum_{y_{1}=0}^{\infty }\sum_{y_{2}=0}^{\infty }f_{\theta }\left(y|\lambda_{t}\right)\left\{\frac{y_{i}}{\lambda_{t,i}}+1+\frac{c|\delta |e^{-c\lambda_{t,i}}\left|u\left(y_{j},\lambda_{t,j}\right)\right|}{\varphi (y,\lambda_{t},\delta )}\right\}\\ & & +\left\{\frac{Y_{t,i}}{\lambda_{t,i}}+1+\frac{c|\delta |e^{-c\lambda_{t,i}}\left|u\left(Y_{t,j},\lambda_{t,j}\right)\right|}{\varphi (Y_{t},\lambda_{t},\delta )}\right\}\\ & \leq & 1+1+\frac{2c\delta_{U}}{\varphi_{L}}+\frac{Y_{t,i}}{\omega_{L}}+1+\frac{2c\delta_{U}}{\varphi_{L}}\\ & = & \frac{Y_{t,i}}{\omega_{L}}+3+\frac{4c\delta_{U}}{\varphi_{L}} \end{array}$$

for (i,j)=(1,2),(2,1) and

$$\begin{array}{ccc} |D_{t,3}\left(\theta \right)| & \leq & \sum_{y_{1}=0}^{\infty }\sum_{y_{2}=0}^{\infty }f_{\theta }\left(y|\lambda_{t}\right)\frac{|u\left(y_{1},\lambda_{t,1}\right)||u\left(y_{2},\lambda_{t,2}\right)|}{\varphi (y,\lambda_{t},\delta )}+\frac{|u\left(Y_{t,1},\lambda_{t,1}\right)||u\left(Y_{t,2},\lambda_{t,2}\right)|}{\varphi (Y_{t},\lambda_{t},\delta )}\\ & = & \frac{8}{\varphi_{L}}. \end{array}$$

The second and fourth parts of the lemma also hold in the same manner. Furthermore, we can show that

$$\begin{array}{ccc} |F_{t,ii}\left(\theta \right)| & \leq & \sum_{y_{1}=0}^{\infty }\sum_{y_{2}=0}^{\infty }f_{\theta }\left(y|\lambda_{t}\right)\left[2\left(1+\alpha \right)\left\{{\left(\frac{y_{i}-\lambda_{t,i}}{\lambda_{t,i}}\right)}^{2}+{\left(\frac{c\delta e^{-c\lambda_{t,i}}u\left(y_{j},\lambda_{t,j}\right)}{\varphi (y,\lambda_{t},\delta )}\right)}^{2}\right\}\right.\\ & & \;\;\;\;\;\left.+\frac{y_{i}}{\lambda_{t,i}^{2}}+\frac{c^{2}\left|\delta \right|e^{-c\lambda_{t,i}}\left|u\left(y_{j},\lambda_{t,j}\right)\right|\left\{1+\left|\delta \right|e^{-y_{i}}\left|u\left(y_{j},\lambda_{t,j}\right)\right|\right\}}{\varphi {\left(y,\lambda_{t},\delta \right)}^{2}}\right]\\ & & +2\alpha \left\{{\left(\frac{Y_{t,i}}{\lambda_{t,i}}-1\right)}^{2}+{\left(\frac{c\delta e^{-c\lambda_{t,i}}u\left(Y_{t,j},\lambda_{t,j}\right)}{\varphi (Y_{t},\lambda_{t},\delta )}\right)}^{2}\right\}\\ & & \;\;\;\;\;-\frac{Y_{t,i}}{\lambda_{t,i}^{2}}-\frac{c^{2}\left|\delta \right|e^{-c\lambda_{t,i}}\left|u\left(Y_{t,j},\lambda_{t,j}\right)\right|\left\{1+\left|\delta \right|e^{-Y_{t,i}}\left|u\left(Y_{t,j},\lambda_{t,j}\right)\right|\right\}}{\varphi {\left(Y_{t},\lambda_{t},\delta \right)}^{2}}\\ & \leq & 2\left(1+\alpha \right)\frac{1}{\omega_{L}}+2\left(1+\alpha \right)\frac{4c^{2}\delta_{U}^{2}}{\varphi_{L}^{2}}+\frac{1}{\omega_{L}}+\frac{2c^{2}\delta_{U}\left(1+2\delta_{U}\right)}{\varphi_{L}^{2}}\\ & & +4\alpha \frac{Y_{t,i}^{2}}{\omega_{L}^{2}}+4\alpha +2\alpha \frac{4c^{2}\delta_{U}^{2}}{\varphi_{L}^{2}}+\frac{Y_{t,i}}{\omega_{L}^{2}}+\frac{2c^{2}\delta_{U}\left(1+2\delta_{U}\right)}{\varphi_{L}^{2}}\\ & = & \frac{4\alpha }{\omega_{L}^{2}}Y_{t,i}^{2}+\frac{1}{\omega_{L}^{2}}Y_{t,i}+\frac{3+2\alpha }{\omega_{L}}+\frac{4c^{2}\delta_{U}\left\{4\left(1+\alpha \right)\delta_{U}+1\right\}}{\varphi_{L}^{2}}+4\alpha \end{array}$$

for (i,j)=(1,2),(2,1),

$$\begin{array}{ccc} |F_{t,33}\left(\theta \right)| & \leq & \alpha \sum_{y_{1}=0}^{\infty }\sum_{y_{2}=0}^{\infty }f_{\theta }\left(y|\lambda_{t}\right){\left\{\frac{u\left(y_{1},\lambda_{t,1}\right)u\left(y_{2},\lambda_{t,2}\right)}{\varphi (y,\lambda_{t},\delta )}\right\}}^{2}\\ & & +\left(1+\alpha \right){\left\{\frac{u\left(Y_{t,1},\lambda_{t,1}\right)u\left(Y_{t,2},\lambda_{t,2}\right)}{\varphi (Y_{t},\lambda_{t},\delta )}\right\}}^{2}\\ & \leq & \frac{16\alpha }{\varphi_{L}^{2}}+\frac{16(1+\alpha )}{\varphi_{L}^{2}}\\ & = & \frac{16(1+2\alpha )}{\varphi_{L}^{2}}, \end{array}$$

$$\begin{array}{ccc} |F_{t,12}\left(\theta \right)| & \leq & \sum_{y_{1}=0}^{\infty }\sum_{y_{2}=0}^{\infty }f_{\theta }\left(y|\lambda_{t}\right)\left[\left(1+\alpha \right)\left\{\frac{y_{1}}{\lambda_{t,1}}+1+\frac{c|\delta |e^{-c\lambda_{t,1}}\left|u\left(y_{2},\lambda_{t,2}\right)\right|}{\varphi (y,\lambda_{t},\delta )}\right\}\right.\\ & & \;\;\;\;\;\;\;\;\;\;\left.\times \left\{\frac{y_{2}}{\lambda_{t,2}}+1+\frac{c|\delta |e^{-c\lambda_{t,2}}\left|u\left(y_{1},\lambda_{t,1}\right)\right|}{\varphi (y,\lambda_{t},\delta )}\right\}+\frac{c^{2}\left|\delta \right|e^{-c(\lambda_{t,1}+\lambda_{t,2})}}{\varphi {\left(y,\lambda_{t},\delta \right)}^{2}}\right]\\ & & +\alpha \left\{\frac{Y_{t,1}}{\lambda_{t,1}}+1+\frac{c|\delta |e^{-c\lambda_{t,1}}\left|u\left(Y_{t,2},\lambda_{t,2}\right)\right|}{\varphi (Y_{t},\lambda_{t},\delta )}\right\}\\ & & \;\;\;\;\;\;\;\;\;\;\times \left\{\frac{Y_{t,2}}{\lambda_{t,2}}+1+\frac{c|\delta |e^{-c\lambda_{t,2}}\left|u\left(Y_{t,1},\lambda_{t,1}\right)\right|}{\varphi (Y_{t},\lambda_{t},\delta )}\right\}+\frac{c^{2}\left|\delta \right|e^{-c(\lambda_{t,1}+\lambda_{t,2})}}{\varphi {\left(Y_{t},\lambda_{t},\delta \right)}^{2}}\\ & \leq & \left(1+\alpha \right)\left\{c^{2}\delta_{U}+1+\left(1+\frac{2c\delta_{U}}{\varphi_{L}}\right)+\left(1+\frac{2c\delta_{U}}{\varphi_{L}}\right)+\frac{4c^{2}\delta_{U}^{2}}{\varphi_{L}^{2}}+\frac{4c\delta_{U}}{\varphi_{L}}+1\right\}\\ & & +\frac{c^{2}\delta_{U}}{\varphi_{L}^{2}}+\alpha \left\{\frac{Y_{t,1}Y_{t,2}}{\omega_{L}^{2}}+\frac{1}{\omega_{L}}\left(1+\frac{2c\delta_{U}}{\varphi_{L}}\right)\left(Y_{t,1}+Y_{t,2}\right)+\frac{4c^{2}\delta_{U}^{2}}{\varphi_{L}^{2}}+\frac{4c\delta_{U}}{\varphi_{L}}+1\right\}\\ & & +\frac{c^{2}\delta_{U}}{\varphi_{L}^{2}}\\ & = & \frac{\alpha }{\omega_{L}^{2}}Y_{t,1}Y_{t,2}+\frac{\alpha }{\omega_{L}}\left(1+\frac{2c\delta_{U}}{\varphi_{L}}\right)\left(Y_{t,1}+Y_{t,2}\right)+4\left(1+2\alpha \right)\frac{c^{2}\delta_{U}^{2}}{\varphi_{L}^{2}}+\frac{2c^{2}\delta_{U}}{\varphi_{L}^{2}}\\ & & +4\left(2+3\alpha \right)\frac{c\delta_{U}}{\varphi_{L}}+\left(1+\alpha \right)c^{2}\delta_{U}+4+5\alpha , \end{array}$$

and

$$\begin{array}{ccc} |F_{t,i3}\left(\theta \right)| & \leq & \sum_{y_{1}=0}^{\infty }\sum_{y_{2}=0}^{\infty }f_{\theta }\left(y|\lambda_{t}\right)\left[\left(1+\alpha \right)\left\{\frac{y_{i}}{\lambda_{t,i}}+1+\frac{c|\delta |e^{-c\lambda_{t,i}}\left|u\left(y_{j},\lambda_{t,j}\right)\right|}{\varphi (y,\lambda_{t},\delta )}\right\}\right.\\ & & \;\;\;\;\;\;\;\;\;\;\;\;\left.\times \frac{|u\left(y_{1},\lambda_{t,1}\right)||u\left(y_{2},\lambda_{t,2}\right)|}{\varphi (y,\lambda_{t},\delta )}+\frac{ce^{-c\lambda_{t,i}}\left|u\left(y_{j},\lambda_{t,j}\right)\right|}{\varphi {\left(y,\lambda_{t},\delta \right)}^{2}}\right]\\ & & +\alpha \left\{\frac{Y_{t,i}}{\lambda_{t,i}}+1+\frac{c|\delta |e^{-c\lambda_{t,i}}\left|u\left(Y_{t,j},\lambda_{t,j}\right)\right|}{\varphi (Y_{t},\lambda_{t},\delta )}\right\}\frac{|u\left(Y_{t,1},\lambda_{t,1}\right)||u\left(Y_{t,2},\lambda_{t,2}\right)|}{\varphi (Y_{t},\lambda_{t},\delta )}\\ & & +\frac{ce^{-c\lambda_{t,i}}\left|u\left(Y_{t,j},\lambda_{t,j}\right)\right|}{\varphi {\left(Y_{t},\lambda_{t},\delta \right)}^{2}}\\ & \leq & \frac{4(1+\alpha )}{\varphi_{L}}\left(1+1+\frac{2c\delta_{U}}{\varphi_{L}}\right)+\frac{2c}{\varphi_{L}^{2}}+\frac{4\alpha }{\varphi_{L}}\left(\frac{Y_{t,i}}{\omega_{L}}+1+\frac{2c\delta_{U}}{\varphi_{L}}\right)+\frac{2c}{\varphi_{L}^{2}}\\ & = & \frac{4\alpha }{\varphi_{L}\omega_{L}}Y_{t,i}+\frac{4c\{1+2\left(1+2\alpha \right)\delta_{U}\}}{\varphi_{L}^{2}}+\frac{4(2+3\alpha )}{\varphi_{L}} \end{array}$$

for (i,j)=(1,2),(2,1). Now, we prove the last two parts of the lemma. Because $F_{t,ij}\left(\theta \right)=\partial D_{t,i}\left(\theta \right)/\partial \lambda_{t,j}$ for i=1,2,3,j=1,2, owing to MVT, it holds that for i=1,2,3,

$$\begin{array}{ccc} |D_{t,i}\left(\theta \right)-{\tilde{D}}_{t,i}\left(\theta \right)| & \leq & \left|\frac{\partial D_{t,i}\left(\theta \right)}{\partial \lambda_{t,1}}{|}_{\lambda_{t}=\lambda_{t}^{*}}\right||\lambda_{t,1}-{\tilde{\lambda }}_{t,1}|+\left|\frac{\partial D_{t,i}\left(\theta \right)}{\partial \lambda_{t,2}}{|}_{\lambda_{t}=\lambda_{t}^{*}}\right|\left|\lambda_{t,2}-{\tilde{\lambda }}_{t,2}\right|\\ & = & \left|F_{t,i1}\left(\theta \right){|}_{\lambda_{t}=\lambda_{t}^{*}}\right||\lambda_{t,1}-{\tilde{\lambda }}_{t,1}|+\left|F_{t,i2}\left(\theta \right){|}_{\lambda_{t}=\lambda_{t}^{*}}\right|\left|\lambda_{t,2}-{\tilde{\lambda }}_{t,2}\right|, \end{array}$$

where $F_{t,ij}\left(\theta \right){|}_{\lambda_{t}=\lambda_{t}^{*}}$ is the same as $F_{t,ij}\left(\theta \right)$ with $\lambda_{t}$ replaced by $\lambda_{t}^{*}$ for j=1,2. Because ${\left(\lambda_{t,i}^{*}\right)}^{-1}\leq \omega_{L}^{-1}$, it can be easily shown that $\left|F_{t,ij}\left(\theta \right){|}_{\lambda_{t}=\lambda_{t}^{*}}\right|$ has the same upper bound as $\left|F_{t,ij}\left(\theta \right)\right|$ by following the aforementioned arguments. Therefore, the lemma is established. □

**Lemma** **A5.**
*Under* ***(A1)*** *–* ***(A3)*** *, we have*

$$\begin{array}{c} E\left(\underset{\theta \in \Theta }{\sup}{∥\frac{\partial^{2}h_{\alpha ,t}\left(\theta \right)}{\partial \theta \partial \theta^{T}}∥}_{1}\right)<\infty \;\;and\;\;E\left(\underset{\theta \in \Theta }{\sup}{∥\frac{\partial h_{\alpha ,t}\left(\theta \right)}{\partial \theta }\frac{\partial h_{\alpha ,t}\left(\theta \right)}{\partial \theta^{T}}∥}_{1}\right)<\infty . \end{array}$$

**Proof.** We can write

$$\begin{array}{c} E\left(\underset{\theta \in \Theta }{\sup}{∥\frac{\partial^{2}h_{\alpha ,t}\left(\theta \right)}{\partial \theta \partial \theta^{T}}∥}_{1}\right)\leq \left(1+\alpha \right)\left\{E\left(\underset{\theta \in \Theta }{\sup}{∥F_{t}\left(\theta \right)∥}_{1}\right)+E\left(\underset{\theta \in \Theta }{\sup}{∥D_{t}\left(\theta \right)\frac{\partial \Lambda_{t}\left(\theta \right)}{\partial \theta^{T}}∥}_{1}\right)\right\}. \end{array}$$

Hence, to show the first part of the lemma, it is sufficient to show that, for i,j=1,2,

$$\begin{array}{ccc} & & E\left(\underset{\theta \in \Theta }{\sup}{∥F_{t,ij}\left(\theta \right)\frac{\partial \lambda_{t,i}}{\partial \theta_{i}}\frac{\partial \lambda_{t,j}}{\partial \theta_{j}^{T}}∥}_{1}\right)<\infty ,\;E\left(\underset{\theta \in \Theta }{\sup}{∥F_{t,i3}\left(\theta \right)\frac{\partial \lambda_{t,i}}{\partial \theta_{i}}∥}_{1}\right)<\infty ,\\ & & E\left(\underset{\theta \in \Theta }{\sup}\left|F_{t,33}\left(\theta \right)\right|\right)<\infty ,\;\mathrm{and}\;E\left(\underset{\theta \in \Theta }{\sup}{∥D_{t,i}\left(\theta \right)\frac{\partial^{2}\lambda_{t,i}}{\partial \theta_{i}\partial \theta_{i}^{T}}∥}_{1}\right)<\infty , \end{array}$$

which can be directly obtained from (iii) of Lemma A1, Lemma A4, and Cauchy–Schwarz inequality. For example,

$$\begin{array}{ccc} & & E\left(\underset{\theta \in \Theta }{\sup}{∥F_{t,12}\left(\theta \right)\frac{\partial \lambda_{t,1}}{\partial \theta_{1}}\frac{\partial \lambda_{t,2}}{\partial \theta_{2}^{T}}∥}_{1}\right)\\ & \leq & {\left\{E{\left(\underset{\theta \in \Theta }{\sup}\left|F_{t,12}\left(\theta \right)\right|\right)}^{2}\right\}}^{1/2}{\left\{E{\left(\underset{\theta \in \Theta }{\sup}{∥\frac{\partial \lambda_{t,1}}{\partial \theta_{1}}\frac{\partial \lambda_{t,2}}{\partial \theta_{2}^{T}}∥}_{1}\right)}^{2}\right\}}^{1/2}\\ & \leq & {\left[E{\left\{C(Y_{t,1}Y_{t,2}+Y_{t,1}+Y_{t,2}+1)\right\}}^{2}\right]}^{1/2}\\ & & \times {\left\{E{\left(\underset{\theta_{1}\in \Theta_{1}}{\sup}{∥\frac{\partial \lambda_{t,1}}{\partial \theta_{1}}∥}_{1}\right)}^{4}\right\}}^{1/4}{\left\{E{\left(\underset{\theta_{2}\in \Theta_{2}}{\sup}{∥\frac{\partial \lambda_{t,2}}{\partial \theta_{2}}∥}_{1}\right)}^{4}\right\}}^{1/4}\\ & < & \infty \end{array}$$

and

$$\begin{array}{ccc} & & E\left(\underset{\theta \in \Theta }{\sup}{∥D_{t,1}\left(\theta \right)\frac{\partial^{2}\lambda_{t,1}}{\partial \theta_{1}\partial \theta_{1}^{T}}∥}_{1}\right)\\ & \leq & {\left\{E{\left(\underset{\theta \in \Theta }{\sup}\left|D_{t,1}\left(\theta \right)\right|\right)}^{2}\right\}}^{1/2}{\left\{E{\left(\underset{\theta_{1}\in \Theta_{1}}{\sup}{∥\frac{\partial^{2}\lambda_{t,1}}{\partial \theta_{1}\partial \theta_{1}^{T}}∥}_{1}\right)}^{2}\right\}}^{1/2}\\ & \leq & {\left[E{\left\{C(Y_{t,1}+1)\right\}}^{2}\right]}^{1/2}{\left\{E{\left(\underset{\theta_{1}\in \Theta_{1}}{\sup}{∥\frac{\partial^{2}\lambda_{t,1}}{\partial \theta_{1}\partial \theta_{1}^{T}}∥}_{1}\right)}^{2}\right\}}^{1/2}\\ & < & \infty . \end{array}$$

The second part of the lemma can be shown in the same manner. □

**Lemma** **A6.**
*Under* ***(A1)*** *–* ***(A3)*** *, we have*

$$\begin{array}{c} \frac{1}{\sqrt{n}}\sum_{t=1}^{n}\underset{\theta \in \Theta }{\sup}{∥\frac{\partial h_{\alpha ,t}\left(\theta \right)}{\partial \theta }-\frac{\partial {\tilde{h}}_{\alpha ,t}\left(\theta \right)}{\partial \theta }∥}_{1}\overset{a.s.}{⟶}0\;\;as\;\;n\to \infty . \end{array}$$

**Proof.** Owing to (iv) and (vi) of Lemma A1 and Lemma A4, we obtain a.s.,

$$\begin{array}{ccc} & & \frac{1}{1+\alpha }\underset{\theta \in \Theta }{\sup}{∥\frac{\partial h_{\alpha ,t}\left(\theta \right)}{\partial \theta }-\frac{\partial {\tilde{h}}_{\alpha ,t}\left(\theta \right)}{\partial \theta }∥}_{1}\\ & \leq & \underset{\theta \in \Theta }{\sup}{∥{\tilde{D}}_{t}\left(\theta \right)∥}_{1}\underset{\theta \in \Theta }{\sup}{∥\Lambda_{t}\left(\theta \right)-{\tilde{\Lambda }}_{t}\left(\theta \right)∥}_{1}+\underset{\theta \in \Theta }{\sup}{∥\Lambda_{t}\left(\theta \right)∥}_{1}\underset{\theta \in \Theta }{\sup}{∥D_{t}\left(\theta \right)-{\tilde{D}}_{t}\left(\theta \right)∥}_{1}\\ & \leq & \left(\sum_{i=1}^{3}\underset{\theta \in \Theta }{\sup}\left|{\tilde{D}}_{t,i}\left(\theta \right)\right|\right)\left(\sum_{i=1}^{2}\underset{\theta_{i}\in \Theta_{i}}{\sup}{∥\frac{\partial \lambda_{t,i}}{\partial \theta_{i}}-\frac{\partial {\tilde{\lambda }}_{t,i}}{\partial \theta_{i}}∥}_{1}\right)\\ & & +\left(\sum_{i=1}^{2}\underset{\theta_{i}\in \Theta_{i}}{\sup}{∥\frac{\partial \lambda_{t,i}}{\partial \theta_{i}}∥}_{1}+1\right)\left(\sum_{i=1}^{3}\underset{\theta \in \Theta }{\sup}\left|D_{t,i}\left(\theta \right)-{\tilde{D}}_{t,i}\left(\theta \right)\right|\right)\\ & \leq & 2C\left(Y_{t,1}+Y_{t,2}+3\right)V\rho^{t}\\ & & +\left(\sum_{i=1}^{2}\underset{\theta_{i}\in \Theta_{i}}{\sup}{∥\frac{\partial \lambda_{t,i}}{\partial \theta_{i}}∥}_{1}+1\right)\times C\left\{Y_{t,1}^{2}+Y_{t,2}^{2}+2Y_{t,1}Y_{t,2}+4\left(Y_{t,1}+Y_{t,2}\right)+6\right\}V\rho^{t}, \end{array}$$

where ${\tilde{D}}_{t}\left(\theta \right)$ and ${\tilde{\Lambda }}_{t}\left(\theta \right)$ are the same as $D_{t}\left(\theta \right)$ and $\Lambda_{t}\left(\theta \right)$ with $\lambda_{t}$ replaced by ${\tilde{\lambda }}_{t}$. Therefore, from Lemma 2.1 in Straumann and Mikosch [45], together with (iii) of Lemma A1, the RHS of the last inequality converges to 0 exponentially fast a.s. and, thus, the lemma is validated. We refer the reader to Straumann and Mikosch [45] and Cui and Zheng [46] for more details on exponentially fast a.s. convergence. □

**Lemma** **A7.**
*Let ${\hat{\theta }}_{\alpha ,n}^{H}={\mathrm{argmin}}_{\theta \in \Theta }H_{\alpha ,n}\left(\theta \right)$. Subsequently, under* ***(A1)*** *–* ***(A3)*** *, we have*

$$\begin{array}{c} {\hat{\theta }}_{\alpha ,n}^{H}\overset{a.s.}{⟶}\theta^{0}\;\;and\;\;\sqrt{n}\left({\hat{\theta }}_{\alpha ,n}^{H}-\theta^{0}\right)\overset{d}{⟶}N\left(0,J_{\alpha }^{-1}K_{\alpha }J_{\alpha }^{-1}\right)\;\;as\;\;n\to \infty . \end{array}$$

**Proof.** As seen in the proof of Theorem 1, $\sup_{\theta \in \Theta }\left|n^{-1}\sum_{t=1}^{n}h_{\alpha ,t}\left(\theta \right)-E\left(h_{\alpha ,t}\left(\theta \right)\right)\right|$ converges to 0 a.s. and $E(h_{\alpha ,t}\left(\theta \right))$ has a unique minimum at $\theta^{0}$. Hence, the first part of the lemma is verified. Next, we handle the second part. Let θ(i), i=1,…,9 be the *i*-th element of θ. Using MVT, we have

$$\begin{array}{c} 0=\frac{1}{\sqrt{n}}\sum_{t=1}^{n}\frac{\partial h_{\alpha ,t}\left(\theta^{0}\right)}{\partial \theta (i)}+\sqrt{n}{\left({\hat{\theta }}_{\alpha ,n}^{H}-\theta^{0}\right)}^{T}\left(\frac{1}{n}\sum_{t=1}^{n}\frac{\partial^{2}h_{\alpha ,t}\left(\theta_{\alpha ,n,i}^{*}\right)}{\partial \theta \partial \theta (i)}\right) \end{array}$$

for some vector $\theta_{\alpha ,n,i}^{*}$ between $\theta_{0}$ and ${\hat{\theta }}_{\alpha ,n}^{H}$, so that, eventually, we can write

$$\begin{array}{c} 0=\frac{1}{\sqrt{n}}\sum_{t=1}^{n}\frac{\partial h_{\alpha ,t}\left(\theta^{0}\right)}{\partial \theta }+\sqrt{n}{\left({\hat{\theta }}_{\alpha ,n}^{H}-\theta^{0}\right)}^{T}\left(\frac{1}{n}\sum_{t=1}^{n}\frac{\partial^{2}h_{\alpha ,t}\left(\theta_{\alpha ,n}^{*}\right)}{\partial \theta \partial \theta^{T}}\right), \end{array}$$

where the term $\partial^{2}h_{\alpha ,t}\left(\theta_{\alpha ,n}^{*}\right)/\partial \theta \partial \theta^{T}$ actually represents a 9×9 matrix whose (i,j)-th entry is $\partial^{2}h_{\alpha ,t}\left(\theta_{\alpha ,n,ij}^{*}\right)/\partial \theta \left(i\right)\partial \theta \left(j\right)$ for some vector $\theta_{\alpha ,n,ij}^{*}$ between $\theta_{0}$ and ${\hat{\theta }}_{\alpha ,n}^{H}$. We first show that

(A1) $$\begin{array}{c} \frac{1}{\sqrt{n}}\sum_{t=1}^{n}\frac{\partial h_{\alpha ,t}\left(\theta^{0}\right)}{\partial \theta }\overset{d}{⟶}N\left(0,K_{\alpha }\right). \end{array}$$

For $\nu ={\left(\nu_{1}^{T},\nu_{2}^{T},\nu_{3}\right)}^{T}\in R^{4}\times R^{4}\times R$, we obtain

$$\begin{array}{ccc} E\left(\nu^{T}\frac{\partial h_{\alpha ,t}\left(\theta^{0}\right)}{\partial \theta }|F_{t-1}\right) & = & \left(1+\alpha \right)\left\{\nu_{1}^{T}\frac{\partial \lambda_{t,1}^{0}}{\partial \theta_{1}}E\left(D_{t,1}\left(\theta^{0}\right)|F_{t-1}\right)\right.\\ & & \left.\;\;\;\;\;\;\;\;\;\;\;+\nu_{2}^{T}\frac{\partial \lambda_{t,2}^{0}}{\partial \theta_{2}}E\left(D_{t,2}\left(\theta^{0}\right)|F_{t-1}\right)+\nu_{3}E\left(D_{t,3}\left(\theta^{0}\right)|F_{t-1}\right)\right\}\\ & = & 0 \end{array}$$

and

$$\begin{array}{c} E{\left(\nu^{T}\frac{\partial h_{\alpha ,t}\left(\theta^{0}\right)}{\partial \theta }\right)}^{2}=\nu^{T}E\left(\frac{\partial h_{\alpha ,t}\left(\theta^{0}\right)}{\partial \theta }\frac{\partial h_{\alpha ,t}\left(\theta^{0}\right)}{\partial \theta^{T}}\right)\nu <\infty \end{array}$$

by Lemma A5. Hence, it follows from the central limit theorem in Billingsley [47] that

$$\begin{array}{c} \frac{1}{\sqrt{n}}\sum_{t=1}^{n}\nu^{T}\frac{\partial h_{\alpha ,t}\left(\theta^{0}\right)}{\partial \theta }\overset{d}{⟶}N\left(0,\nu^{T}K_{\alpha }\nu \right), \end{array}$$

which implies (A1). Now, we claim that

(A2) $$\begin{array}{c} -\frac{1}{n}\sum_{t=1}^{n}\frac{\partial^{2}h_{\alpha ,t}\left(\theta_{\alpha ,n,ij}^{*}\right)}{\partial \theta (i)\partial \theta (j)}\overset{a.s.}{⟶}J_{\alpha }^{ij}, \end{array}$$

where $J_{\alpha }^{ij}$ denotes the (i,j)-th entry of $J_{\alpha }$. From Lemma A5, $J_{\alpha }$ is finite. Further, after some algebras, we have

$$\begin{array}{ccc} & & \nu^{T}\left(-J_{\alpha }\right)\nu \\ & = & \left(1+\alpha \right)E\left[\sum_{y_{1}=0}^{\infty }\sum_{y_{2}=0}^{\infty }f_{\theta^{0}}^{1+\alpha }\left(y|\lambda_{t}\right)\left\{\left(\nu_{1}^{T}\frac{\partial \lambda_{t,1}^{0}}{\partial \theta_{1}}\right)\left(\frac{y_{1}}{\lambda_{t,1}^{0}}-1+\frac{c\delta^{0}e^{-c\lambda_{t,1}^{0}}u\left(y_{2},\lambda_{t,2}^{0}\right)}{\varphi (y,\lambda_{t}^{0},\delta^{0})}\right)\right.\right.\\ & & \left.\left.\;\;\;\;\;\;\;\;\;\;\;\;\;\;\;\;\;\;\;+\left(\nu_{2}^{T}\frac{\partial \lambda_{t,2}^{0}}{\partial \theta_{2}}\right)\left(\frac{y_{2}}{\lambda_{t,2}^{0}}-1+\frac{c\delta^{0}e^{-c\lambda_{t,2}^{0}}u\left(y_{1},\lambda_{t,1}^{0}\right)}{\varphi (y,\lambda_{t}^{0},\delta^{0})}\right)\right.\right.\\ & & \left.{\left.\;\;\;\;\;\;\;\;\;\;\;\;\;\;\;\;\;\;\;+\nu_{3}\frac{u\left(y_{1},\lambda_{t,1}^{0}\right)u\left(y_{2},\lambda_{t,2}^{0}\right)}{\varphi (y,\lambda_{t}^{0},\delta^{0})}\right\}}^{2}\right]>0 \end{array}$$

by (v) of Lemma A1, which implies that $J_{\alpha }$ is non-singular. Note that we can write

$$\begin{array}{ccc} & & \left|\frac{1}{n}\sum_{t=1}^{n}\frac{\partial^{2}h_{\alpha ,t}\left(\theta_{\alpha ,n,ij}^{*}\right)}{\partial \theta (i)\partial \theta (j)}-E\left(\frac{\partial^{2}h_{\alpha ,t}\left(\theta^{0}\right)}{\partial \theta (i)\partial \theta (j)}\right)\right|\\ & \leq & \underset{\theta \in \Theta }{\sup}\left|\frac{1}{n}\sum_{t=1}^{n}\frac{\partial^{2}h_{\alpha ,t}\left(\theta \right)}{\partial \theta (i)\partial \theta (j)}-E\left(\frac{\partial^{2}h_{\alpha ,t}\left(\theta \right)}{\partial \theta (i)\partial \theta (j)}\right)\right|+\left|E\left(\frac{\partial^{2}h_{\alpha ,t}\left(\theta_{\alpha ,n,ij}^{*}\right)}{\partial \theta (i)\partial \theta (j)}\right)-E\left(\frac{\partial^{2}h_{\alpha ,t}\left(\theta^{0}\right)}{\partial \theta (i)\partial \theta (j)}\right)\right|. \end{array}$$

Because $\partial^{2}h_{\alpha ,t}\left(\theta \right)/\partial \theta \left(i\right)\partial \theta \left(j\right)$ is stationary and ergodic, from Lemma A5, the first term on the RHS of the inequality converges to 0 a.s. Moreover, the second term converges to 0 by the dominated convergence theorem. Hence, (A2) is asserted. Therefore, from (A1) and (A2), the second part of the lemma is established. □

**Proof of Theorem 2.** From MVT, we get

$$\begin{array}{c} \frac{1}{n}\sum_{t=1}^{n}\frac{\partial h_{\alpha ,t}\left({\hat{\theta }}_{\alpha ,n}^{H}\right)}{\partial \theta (i)}-\frac{1}{n}\sum_{t=1}^{n}\frac{\partial h_{\alpha ,t}\left({\hat{\theta }}_{\alpha ,n}\right)}{\partial \theta (i)}={\left({\hat{\theta }}_{\alpha ,n}^{H}-{\hat{\theta }}_{\alpha ,n}\right)}^{T}\left(\frac{1}{n}\sum_{t=1}^{n}\frac{\partial^{2}h_{\alpha ,t}\left(\zeta_{\alpha ,n,i}\right)}{\partial \theta \partial \theta (i)}\right) \end{array}$$

for some vector $\zeta_{\alpha ,n,i}$ between ${\hat{\theta }}_{\alpha ,n}^{H}$ and ${\hat{\theta }}_{\alpha ,n}$. Thus, we can write

$$\begin{array}{c} \frac{1}{n}\sum_{t=1}^{n}\frac{\partial h_{\alpha ,t}\left({\hat{\theta }}_{\alpha ,n}^{H}\right)}{\partial \theta }-\frac{1}{n}\sum_{t=1}^{n}\frac{\partial h_{\alpha ,t}\left({\hat{\theta }}_{\alpha ,n}\right)}{\partial \theta }={\left({\hat{\theta }}_{\alpha ,n}^{H}-{\hat{\theta }}_{\alpha ,n}\right)}^{T}\left(\frac{1}{n}\sum_{t=1}^{n}\frac{\partial^{2}h_{\alpha ,t}\left(\zeta_{\alpha ,n}\right)}{\partial \theta \partial \theta^{T}}\right), \end{array}$$

where the (i,j)-th entry of $\partial^{2}h_{\alpha ,t}\left(\zeta_{\alpha ,n}\right)/\partial \theta \partial \theta^{T}$ is $\partial^{2}h_{\alpha ,t}\left(\zeta_{\alpha ,n,ij}\right)/\partial \theta \left(i\right)\partial \theta \left(j\right)$ for some vector $\zeta_{\alpha ,n,ij}$ between ${\hat{\theta }}_{\alpha ,n}^{H}$ and ${\hat{\theta }}_{\alpha ,n}$. Since $n^{-1}\sum_{t=1}^{n}\partial h_{\alpha ,t}\left({\hat{\theta }}_{\alpha ,n}^{H}\right)/\partial \theta =0$ and $n^{-1}\sum_{t=1}^{n}\partial {\tilde{h}}_{\alpha ,t}\left({\hat{\theta }}_{\alpha ,n}\right)/\partial \theta =0$, we have

$$\begin{array}{c} \frac{1}{\sqrt{n}}\sum_{t=1}^{n}\frac{\partial {\tilde{h}}_{\alpha ,t}\left({\hat{\theta }}_{\alpha ,n}\right)}{\partial \theta }-\frac{1}{\sqrt{n}}\sum_{t=1}^{n}\frac{\partial h_{\alpha ,t}\left({\hat{\theta }}_{\alpha ,n}\right)}{\partial \theta }=\sqrt{n}{\left({\hat{\theta }}_{\alpha ,n}^{H}-{\hat{\theta }}_{\alpha ,n}\right)}^{T}\left(\frac{1}{n}\sum_{t=1}^{n}\frac{\partial^{2}h_{\alpha ,t}\left(\zeta_{\alpha ,n}\right)}{\partial \theta \partial \theta^{T}}\right). \end{array}$$

The LHS of the above equation converges to 0 a.s. by Lemma A6, and $n^{-1}\sum_{t=1}^{n}\partial^{2}h_{\alpha ,t}\left(\zeta_{\alpha ,n}\right)$/$\partial \theta \partial \theta^{T}$ converges to $-J_{\alpha }$ a.s. in a similar way as in the proof of Lemma A7. Therefore, the theorem is established due to Lemma A7. □
